# Nursing competencies for family‐centred care in the hospital setting: A multinational Q‐methodology study

**DOI:** 10.1111/jan.14719

**Published:** 2020-12-13

**Authors:** Bram Hengeveld, Jolanda M. Maaskant, Robert Lindeboom, Andrea P. Marshall, Hester Vermeulen, Anne M. Eskes

**Affiliations:** ^1^ Livio Enschede the Netherlands; ^2^ Vilans Utrecht the Netherlands; ^3^ Emma Children’s Hospital Amsterdam UMC, University of Amsterdam Amsterdam the Netherlands; ^4^ Department of Clinical Epidemiology, Biostatistics and Bioinformatics Amsterdam UMC, University of Amsterdam Amsterdam the Netherlands; ^5^ Menzies Health Institute Queensland School of Nursing and Midwifery Griffith University Southport Queensland Australia; ^6^ Gold Coast Health Southport Queensland Australia; ^7^ Scientific Center for Quality of Healthcare (IQ healthcare) Radboud University Medical Center, Radboud Institute for Health Sciences Nijmegen the Netherlands; ^8^ Faculty of Health and Social Studies HAN University of Applied Sciences Nijmegen The Netherlands; ^9^ Department of Surgery Amsterdam UMC, University of Amsterdam Amsterdam the Netherlands

**Keywords:** clinical competence, education, nursing, factor analysis, statistical, family nursing, nurses, nursing, qualitative research, stakeholder participation

## Abstract

**Aim:**

to identify: (1) nursing competencies for FCC in a hospital setting; and (2) to explore perspectives on these competencies among Dutch and Australian professionals including lecturers, researchers, Registered Nurses and policy makers.

**Design:**

A multinational cross‐sectional study using Q‐methodology.

**Methods:**

First, an integrative review was carried out to identify known competencies regarding FCC and to develop the Q‐set (search up to July 2018). Second, purposive sampling was used to ensure stakeholder involvement. Third, participants sorted the Q‐set using a web‐based system between May and August 2019. Lastly, the data were analysed using a by‐person factor analysis. The commentaries on the five highest and lowest ranked competencies were thematically analysed.

**Results:**

The integrative review identified 43 articles from which 72 competencies were identified. In total 69 participants completed the Q‐sorting. We extracted two factors with an explained variance of 24%. The low explained variance hampered labelling. Based on a post‐hoc qualitative analysis, four themes emerged from the competencies that were considered most important, namely: (a) believed preconditions for FCC; (b) promote a partnership between nurses, patients and families; (c) be a basic element of nursing; and (d) represent a necessary positive attitude and strong beliefs of the added value of FCC. Three themes appeared from the competencies that were considered least important because they: (a) were not considered a specific nursing competency; (b) demand a multidisciplinary approach; or (c) require that patients and families take own responsibility.

**Conclusions:**

Among healthcare professionals, there is substantial disagreement on which nursing competencies are deemed most important for FCC.

**Impact:**

Our set of competencies can be used to guide education and evaluate practicing nurses in hospitals. These findings are valuable to consider different views on FCC before implementation of new FCC interventions into nursing practice.

## 摘要

**目的:**

确定: (1) 医院环境中FCC的护理能力; (2) 探讨荷兰和澳大利亚专业人员 (包括讲师、研究者、注册护士和决策者) 对这些能力的看法。

**设计:**

采用Q方法的跨国横断面研究。

**方法:**

首先进行综合评估, 以确定与FCC有关的已知能力, 并制定Q集 (检索截至2018年7月) 。第二, 采用目的性抽样来确保利益相关者参与度。第三, 参与者在2019年5月至8月期间使用基于网络 (web)的系统对Q集进行排序。最后, 采用个人因素分析法对数据进行分析。对排名最高和最低的5项能力的评注进行了专题分析。

**结果:**

在综合评估中, 研究了43篇文章, 从中确定了72项能力。共有69名参与者完成了Q分类。我们提取了两个因素, 解释方差为24%。低解释方差阻碍了标签化。基于事后定性分析, 从被视为是最重要的能力中衍生出四个主题, 即: (a) 所信的FCC先决条件; (b) 促进护士、患者和家属之间的合作关系; (c) 成为护理工作的基本要素; (d) 代表一种必要的积极态度和对FCC附加价值的强烈信念。从被视为是最不重要的能力中衍生出三个主题, 因为它们: (a) 没有被视为是一种特定的护理能力; (b) 需要采用多学科方法; 或 (c) 要求患者和家属自行承担责任。

**结论:**

在医疗保健专业人员中, 对于哪些护理能力被视为对FCC而言最重要, 存在很大的分歧。

**影响:**

我们的一系列能力可以用来指导教育和评估医院的执业护士。在护理实践中实施新的FCC干预措施之前, 在考虑不同的FCC观点方面, 这些研究结果很有价值。

## 1 INTRODUCTION

Family‐centred care (FCC) has received increased attention in recent years outside of paediatric nursing, from which it originated (Cene et al., 2016). In paediatric wards the involvement of family members is almost standard; however, for hospitalized adults, care is shifting slowly to a more family‐centred approach. Nevertheless, the increased attention towards FCC in adult in‐hospital care can be seen in a wide range of settings, patients and areas of attention, for example in stroke care, (Forster et al., 2013; Lindley et al., 2017), surgery, (Schreuder et al., 2019) and intensive care, (Bench et al., 2015; Op't Hoog, Dautzenberg, Eskes, Vermeulen, & Vloet, 2020). Although consensus on a definition of FCC is lacking, there is agreement on the core principles of FCC which include unbiased communication, collaboration in care and/or decision‐making and recognition of expertise (Banerjee et al., 2018; Kuo et al., 2012; Lor et al., 2016; Mikkelsen & Frederiksen, 2011). FCC aims to support the establishment of a mutual partnership and collaboration among nurses, patients and their family caregivers in a way that promotes patient satisfaction and self‐determination (Kitson et al., 2014). However, misunderstandings about the process of FCC can drive families and healthcare workers further apart (Kuo et al., 2012).

## 2 BACKGROUND

A minimum level of education among healthcare professionals can be seen as a prerequisite to provide adequate patient‐ and family‐centred care. This is particularly important for nurses, as they provide approximately 70% of all in‐hospital care (van Oostveen et al., 2015) and are considered to have an essential role in adopting a culture of FCC. Although FCC is an expected approach worldwide in the delivery of healthcare, it is known that families’ needs are not always met (Anker‐Hansen et al., 2018; Hirakawa et al., 2011; Smith & Kendal, 2018; Ventura et al., 2014). Incorporating FCC competencies in undergraduate and postgraduate education is needed to support the shift towards a more family‐centred environment (Philibert et al., 2011). Education underpins clinical practice and professional behaviour and is essential for widespread implementation of FCC (Tan et al., 2018; Wensing et al., 2013). Yet, competencies specific to FCC are not clearly articulated in the literature. Given the importance of nurses in implementing FCC, explicating nursing competencies and understanding nurses’ perceptions of these competencies can support improved delivery of FCC in daily practice, guide educational curricula and inform future research.

## 3 THE STUDY

### 3.1 Aims

The aim of this study was to identify: (1) nursing competencies for FCC in a hospital setting; and (2) to explore perspectives on these competencies among Dutch and Australian professionals including lecturers, researchers, registered nurses and policy makers.

### 3.2 Design

To develop a ranked set of nursing competencies for FCC in hospital and identify themes in professionals’ opinions on their relative importance, we conducted a cross‐sectional study in the Netherlands and Australia using Q‐methodology. Q‐methodology combines quantitative and qualitative analyses in a four‐phase process to reveal subjective viewpoints among groups of participants and identify underlying explanatory variables in the dataset called ‘factors’ (Watts & Stenner, 2012). In a Q‐study, researchers ask participants to rank a pre‐specified set of statements, based on their views on the subject and to explain their choices. This method fits our purpose as views on the importance of competencies are inherently subjective.

This study is reported according to applicable criteria of the Strengthening the Reporting of Observational Studies in Epidemiology (STROBE) statement (von Elm et al., 2008) and complemented with relevant criteria of the Preferred Reporting Items for Systematic Reviews and Meta‐Analyses (PRISMA) statement, (Moher et al., 2015; Shamseer et al., 2015) and Assessment and Review Instrument for Q‐methodology (ARIQ) (Dziopa & Ahern, 2011).

### 3.3 Phase 1 – Development of the Q‐set: an integrative review

#### 3.3.1 Validity, reliability and rigour

We carried out an integrative review of the literature to identify existing competencies regarding FCC. All types of studies that described nursing competencies for FCC (as a subject of the publication) in title and/or abstract were eligible for inclusion. If the title and or abstract made it clear that competencies for FCC were available in the publication, the full text was also screened. Our search included the databases MEDLINE (Pubmed), CINAHL, ERIC and the Cochrane Database of Systematic Reviews. We used the keywords nursing, clinical competence, competency, family‐centred care, case management, family caregivers and variation on these topics (See Supplementary file S1). Studies published in Dutch or English were eligible for inclusion. No restriction was placed on the year of publication. We included studies conducted up to 5 July 2018.

One of the authors (BH) screened titles and abstracts using an online tool for systematic reviews (Rayyan https://rayyan.qcri.org). Full‐text versions of articles were obtained if they matched the eligibility criteria or if further scrutiny was needed regarding eligibility. Afterwards, two authors (AE and BH) independently extracted competencies from the included articles using an Excel spreadsheet, specially designed for this study. First author and year of publication were extracted and the exact wording of the competencies as mentioned in the publication. After extraction, the competencies were compared and discussed. First, an overview of identical competencies as extracted by the two authors was made. Lack of consensus regarding the remaining competencies was resolved through discussions, assisted by a third author (HV) if necessary.

After agreement on the competencies to be included was reached, BH categorized the competencies using key concepts described in the CanMEDS framework. The CanMEDS framework comprises seven domains: medical expert, communicator, collaborator, leader, health advocate, scholar and professional and describes abilities that healthcare professionals require to meet the needs of patients to whom they provide care. (Frank & Royal College of Physicians and Surgeons of Canada, 2005) For the purpose of this study the CanMEDS domain ‘medical expert’ was converted into ‘nursing expert’. After categorization, BH and AE screened all grouped competencies for overlapping themes and combined competencies which were conceptually similar to reduce the number. This resulted in a final set (‘Q‐set’) of competencies which was used in phase 3 (the Q‐sort). To ensure usability of the competencies in the Q‐sort, the English list was checked by one of the authors (AM), a native English speaker. The final list of English competencies was translated to Dutch using a forward‐backward translation procedure, meeting the ISO 17100:2015 standards, the European certificate for translator services.

### 3.4 Phase 2 – Development of the P‐set: selecting participants

#### 3.4.1 Participants

Participants were eligible if they were willing to participate and able to proficiently read and write in Dutch or English, were 18 years or older and from the Netherlands or Australia. Also, they had to belong to one of the four groups: registered nurses, policy makers (e.g. directors in hospital care), researchers with peer‐reviewed publications on FCC, or nationally recognized experts on FCC. While recommendations for sample size in Q‐studies differs, most suggest a number of participants equal to the number of statements (Dziopa & Ahern, 2011). We therefore aimed for a minimum of 72 participants.

An invitation was sent by e‐mail where a detailed description of the study was given. We aimed to include an equal number of participants from each of the four groups. Reminders were sent if participants did not participate within two weeks (Edwards et al., 2009). The following baseline characteristics of the participants were collected: age, gender, current clinical setting, job description, highest level of education, number of years of experience in their current position, total number of working hours and hours spent on direct patient care, lecturing, research and policy making.

### 3.5 Phase 3 ‐ Using the Q‐sort: sorting the competencies

#### 3.5.1 Appraising the importance of the competencies in the Q‐sort

The participants ranked the competencies in order of importance using a specially designed website (accessible via https://qsort.family‐centeredcare.com, version weas available in Dutch and English). The ranking process started with the division of the competencies into three categories (i.e. most important, least important and neutral). In the second step, the participants placed the competencies in the Q‐sort. Our Q‐sort was a quasi‐normal shaped symmetrical table with 11 columns (See Supplementary file S2). Each column represented a score ranging from 1‐11 on the importance of a competency, with 1 being least important. Participants were forced to give a ranking of importance to the competencies. In the third step, each participant was shown an overview of their Q‐sort with the ability to change their ranking. If they agreed with the ranking, they were asked to give a short rationale for their choice of most and least important competencies. These comments were used for qualitative analysis of our data. The created Q‐sort was pilot tested in a convenience sample of three members of the target population. No major adjustments after pilot testing were needed.

### 3.6 Data collection

Data were acquired and stored on an internet server running HTMLQ. Each user received a unique URL via e‐mail. The code in the URL was used to save the data in a separate CSV‐file for each participant. The website is hosted at Mijndomein.nl (https://www.mijndomein.nl) and fully complied with the General Data Protection Regulation (GDPR).

### 3.7 Phase 4 ‐ Data analysis

#### 3.7.1 Quantitative analysis

To identify factors in our participants’ viewpoints on the competencies, the Q‐sorts were analysed using by‐person factor analysis. Factors were extracted using centroid factor analysis. We used the following criteria for a factor to be extracted (Watts & Stenner, 2012):

- Horn's parallel analysis. The observed Eigenvalues should exceed the 95th percentile of Eigenvalues generated using 1,000 random data sets of equal size. (Horn, 1965; O'Connor, 2000)
- The factor should have at least two significant factor loadings (*p* < .01) (Watts & Stenner, 2012)
- ‘Humphrey's rule’ (Watts & Stenner, 2012): the cross product of the two highest loadings of the factor exceeds twice the standard error of the study. In case of 72 items in the Q‐sort the standard error is 1/√72 = 0.118.

After factor extraction, orthogonal rotation applying the varimax technique was used for alignment of factors which maximizes the number of factor loadings for the Q‐sorts. Next, Q‐sorts with factor loadings of 0.6 on any one factor and no more than 0.4 on any other factor were used to construct factor estimates. Factor estimates were constructed using weighted averages of Q‐sorts within a factor. We also examined whether Q‐sorts loaded significantly on more than 1 factor (‘confounded Q‐sorts’), to exclude them from the creation of factor estimates.

Next, z‐scores of the weighted scores were calculated to enable comparisons between factors. A competency whose z‐score within a factor differs significantly (*p* < .05) from its z‐scores in all other factors is a ‘distinguishing’ competency for that factor. Distinguishing competencies are pivotal in the interpretation of factors, as they signal unique viewpoints on competencies for family‐centred care.

Normality of scores for individual data was investigated using histograms and the Shapiro‐Wilks test with a correction for multiple testing using the Holms step‐down method (Holm, 1979). Based on normality we either calculated means and standard deviations or median and inter quartile ranges of all participants for each competency to create an overall rank of competencies.

We used Ken‐Q Analysis Desktop Edition (KADE) 1.0.1 (https://github.com/shawnbanasick/kade), a program specifically designed for the analysis of Q‐studies (Banasick, 2019), and IBM SPSS Statistics for Windows, Version 25.0. (Armonk, NY: IBM Corp) for analysis of the data.

#### 3.7.2 Quantitative post hoc analysis

In case of low explained variance, several explorative subgroup analyses are planned based on the two extracted factors in the initial analysis. These subgroups were defined as follow:

- By country (Australia and the Netherlands).
- By professional group defined as frontline healthcare professionals (i.e. nurses) and non‐frontline healthcare professionals (i.e. lectures, policy makers and researchers).
- By professional group separately (i.e. lecturers, researchers, registered nurses and policy makers).
- By professional groups via the leave‐one out method. In each step another professional group is removed from analysis.

To be able to compare the explained variance to our total data set, we only counted the explained variance in the two factors with the highest Eigenvalues.

#### 3.7.3 Qualitative post hoc analysis

The commentaries on the five highest and lowest ranked competencies were analysed using thematic analysis methodology. Data were coded independently by two researchers (AE and JM). This process involved the identification of recurrent issues by reading the transcripts in an iterative way. The results of the two researchers were compared and discussed until consensus was reached. After completing the initial coding patterns were discussed and preliminary themes were defined. Thereafter, both researchers went back and forth between the transcripts, codes and themes until a set of coherent and meaningful themes was agreed upon. The themes were finalized after presentation and discussion in the research team.

### 3.8 Ethical considerations

The Medical Ethics Review Committee of the Amsterdam UMC (Amsterdam UMC, University of Amsterdam, Amsterdam) reviewed the study protocol and concluded that the Medical Research Involving Human Subject Act (WMO) does not apply to this study (reference number W17_067 #17.085). Therefore, official approval of this project by a Medical Ethics Review Committee in the Netherlands was not required. The office for research ethics of the Griffith university provided full ethical clearance (GU Ref No: 2019/273). Consent to participate in this project was implied by participants contribution to data collection. Participants were allowed to leave the study at any time for any reason if they wish to do so, without any consequences.

## 4 RESULTS

### 4.1 Review of the literature

**Figure 1:**
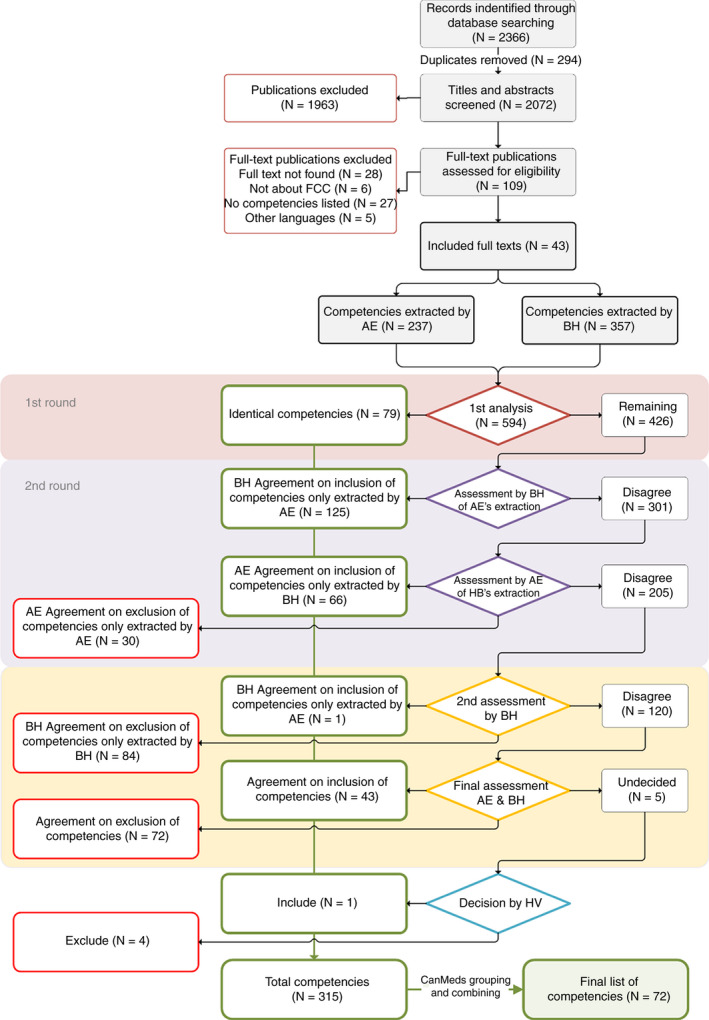
Adapted PRISMA flowchart of phase 1 of the study (development of Q‐set) [Colour figure can be viewed at wileyonlinelibrary.com]

The initial combined search yielded 2,366 hits of which 43 full‐text publications were included in the review (see Figure 1: flowchart of the process; Supplementary file S3: List of included publications in integrative review). BH and AE independently extracted 594 competencies, using a specially designed Excel spreadsheet for collecting and combining competencies. After deliberation, a total of 315 competencies were agreed upon. After grouping and combining using the CanMEDS key concepts, 96 competencies remained. JM and AM screened the competencies for further overlap and reduced the final number of competencies to 72. The final set of competencies can be found in Supplementary file S4.

### 4.2 Participants

We invited 89 participants: 46 from Australia; and 43 from the Netherlands. Sixty‐nine people (77.5%) completed the online Q‐sort between May and August 2019 of whom 35 were from Australia and 34 from the Netherlands. Most participants was female (*N* = 54; 78.3%). Participants had a mean age of 46.7 years. Of the participating nurses, lecturers and researchers and policymakers respectively 38.9%, 33.3%, 52.2% and 76.9% were Dutch. Baseline characteristics of the participants can be found in Table 1.

**Table 1:** Characteristics of participants

	Total	Nurses	Lecturers	Researchers	Policy makers
*N* (%)	69 (100)	18 (26.1)	15 (21.7)	23 (33.3)	13 (18.8)
Dutch *N* (%)	34 (49.3)	7 (38.9)	5 (33.3)	12 (52.2)	10 (76.9)
Gender *N* (%)					
Female	54 (78.3)	15 (27.8)	10 (18.5)	19 (35.2)	10 (18.5)
Male	7 (10.2)	1(14.3)	1(14.3)	2 (28.6)	3 (42.8)
Non‐binary	1 (1.5)		1 (100)		
Rather not state	2 (2.9)		1 (50)	1 (50)	
Missing	5 (7.2)	2 (40)	2 (40)	1 (20)	
Age (yrs) mean (*SD*)	46.7 (10.3)	39.6 (12.0)	44.9 (8.7)	51.6 (7.6)	47.6 (9.5)
Level of education *N* (%)					
Vocational	1		1(100)		
Bachelor	11	7 (63.6)			4 (36.4)
Master	26	7 (26.9)	8 (30.8)	3 (11.5)	8 (30.8)
PhD	28	3 (10.7)	4 (14.3)	20 (71.4)	1 (3.6)
Missing	3	1 (33.3)	2 (66.7)		
Experience in current position (yrs) median (IQR)^a^		0.5 (7)	3.5 (8.75)	1.5 (13.75)	2 (8)

^a^ Medians and IQR are given, data were not normally distributed (Shapiro–Wilks *p* < .001)

### 4.3 Sorted competencies and extracted factors

Two factors were extracted, with 53 (76.8%) Q‐sorts loading significantly on a factor. A third factor failed to meet the requirement of an Eigenvalue higher than the 95th percentile of 1.000 randomly generated Eigenvalues based on parallel analysis. After varimax rotation the two factors had an explained variance of 24%.

Characteristics for the two factors, confounded Q‐sorts, non‐significant Q‐sorts and distribution of factors within professions are listed in Table 2. The competencies’ overall ranking and ranking within the extracted factors can be found in Table 3. Based on the low explained variance of the factors (i.e. 24%) we decided that advancing with meaningful labelling and a detailed description of the factors would not add value. The complete ranked set of competencies can be found in Table 3.

**Table 2:** Characteristics of extracted factors

Factor	Q‐sorts loading on factor (*n*)^a^	Eigen‐value	Humphrey's rule^b^	Explained variance	Australian *N* (%)	Dutch *N* (%)	Nurses *N* (%)	Lecturers *N* (%)	Researchers *N* (%)	Policy makers *N* (%)
1	28	12.9	0.52	13	12 (42.9)	16 (57.1)	11 (39.3)	2 (7.1)	9 (32.1)	6 (21.4)
2	25	3.6	0.40	11	16 (64.0)	9 (36.0)	5 (20.0%)	8 (32.0)	8 (32.0)	4 (16.0)
Confounded Q‐sorts^c^	0									
Non‐significant Q‐sorts	14				7 (50.0)	7 (50.0)	2 (14.3)	5 (35.7)	4 (28.6)	2 (14.3)

^a^ Only Q‐sort loading significantly (*p* < .01) are counted for Factors.

^b^ The cross product of the two highest loadings of the factor should exceed twice the standard error of 0.118.

^c^ Confounded Q‐sorts load significantly on more than one factor.

**Table 3:** List of competencies ranked in order of importance

Competency (the nurse…)	CanMeds Role	Overall rank	Mean score (1–11)	*SD*	Highest rank	Lowest rank
28. Acknowledges patients and family members as the source of control and full partner in providing compassionate and coordinated care based on respect for patients’ preferences, values, needs and family members’ expertise.	Collaborator	1	8,38^a^	2,47	11	1
35. Supports patients and family members to participate in decision making regarding care, at the level with which they are comfortable.	Collaborator	2	8,14	2,12	11	1
21. Communicates in an honest, compassionate, non‐judgmental and calm manner to family members	Communicator	3	7,86	2,15	11	2
1. Identifies and responds to the needs of patients and family members.	Nursing Expert	4	7,71	2,34	11	2
32. Promotes, guides and monitors active participation of family members in care for patients in accordance with preferences of patients and family members.	Collaborator	5	7,67	2,24	11	2
56. Enhances or reinforces the patients’ and family members’ senses of autonomy and self‐determination through education and support to maintain their sense of control and quality of life	Health Advocate	6	7,65^b^	2,59	11	2
22. Provides appropriate and timely information to patients and family members to facilitate understanding and support informed decision making	Communicator	7	7,46	2,09	11	3
20. Provides coherent and congruent information in easily understood language to keep the family members informed about diagnoses, treatments, progress, prognosis and transfers.	Communicator	8	7,19	2,22	11	1
13. Prioritizes goals to achieve the outcomes deemed most important by patients and family members	Communicator	9	7,17	2,18	11	1
33. Collaborates with all members of the healthcare team to facilitate the provision of physical and emotional care and support to patients and family members	Collaborator	10	7,12	2,29	11	3
29. Assesses family members’ preferred level of participation and role in decision making	Collaborator	11	7,04	2,08	11	1
31. Promotes family presence in accordance with patient preferences	Collaborator	12	7,00	2,19	11	1
26. Establishes and maintains a therapeutic relationship with patients and family members.	Communicator	13	6,91	2,53	11	1
9. Acknowledges the experiences, emotions, concerns and needs of family members through authentic conversation	Communicator	14	6,83	2,11	11	2
3. Anticipates the needs of, and care for patients and family members	Nursing Expert	15	6,80	2,42	11	2
23. Discusses communication preferences with patients and family members	Communicator	16	6,77	2,11	11	2
18. Assesses family members’ current knowledge, received information and experience of family members regarding patients' diagnoses, treatments and prognosis.	Communicator	17	6,74	2,08	11	1
36. Enables the mutual exchange of information among patients, family members and healthcare professionals	Collaborator	18	6,74	2,21	11	3
46. Identifies vulnerable families and adapts the care environment to facilitate family presence and involvement	Health Advocate	19	6,72	2,04	11	1
10. Provides emotional and psychosocial support to family members	Communicator	20	6,70	2,26	11	3
53. Empowers family members to make their own choices, solve problems and promote self‐help and caring abilities	Health Advocate	21	6,68	2,31	11	2
12. Listens to, encourages construction of, and documents care goals in collaboration with patients and family members	Communicator	22	6,65	2,13	10	1
43. Promotes a patient‐ and family‐centred care environment for ethical decision‐making and advocacy for patients	Leader	23	6,55	2,60	11	1
37. Informs family members accurately and honestly in response to their questions, but also without being asked.	Collaborator	24	6,45	2,30	11	1
66. Admits when one's own knowledge and understanding fall short and seeks additional resources to provide care in a manner that respect the dignity and cultural integrity of patients and family members.	Scholar	25	6,39	2,46	11	1
8. Supports family members in coping with the psychosocial aspects of illness, based on their needs, healthcare literacy and individual situation	Communicator	26	6,39	2,54	11	1
25. Demonstrates respect for coping strategies and cultural and religious preferences and practices of patients and family members when discussing options, particularly when families decline evidence‐based therapy	Communicator	27	6,33	2,10	11	1
19. Uses a range of strategies to communicate with family members, including reading, writing, speaking, validating, listening, teaching, and eliciting the stories of family members	Communicator	28	6,32	2,35	11	2
47. Advocates on behalf of patients and family members to promote coordinated service delivery	Health Advocate	29	6,28	2,27	11	1
24. Provides care beyond technical‐oriented tasks to connect with patients and family members in meaningful ways on a personal level.	Communicator	30	6,23	2,62	11	2
54. Supports patients and family members and reinforces their ability to accept the illness and regain control, regardless of prognosis	Health Advocate	31	6,19	2,44	11	1
48. Advocates for confidentiality and privacy for patients and family members	Health Advocate	32	6,13	2,28	11	1
41. Educates and coaches patients, families and health professionals to facilitate family‐centred care practices.	Leader	33	6,07	1,91	10	2
17. Assesses family members’ health literacy and readiness to learn.	Communicator	34	6,06	2,17	11	1
40. Identifies and interprets barriers to the delivery of family‐centred care within the healthcare setting and develop strategies to resolve these issues	Leader	35	6,04	2,42	11	1
50. Understands the impact of illness on families and vice versa	Health Advocate	36	6,01	2,30	11	1
7. Assesses and evaluates the ability of families to deliver appropriate and safe care	Nursing Expert	37	5,96	2,32	10	1
69. Receives feedback from family members and develops actions based on that feedback.	Scholar	38	5,94	1,99	11	2
5. Uses a family‐centred approach to minimize the risk of harm to patients and family members	Nursing Expert	39	5,93	2,32	11	1
30. Engages family members in active partnerships that promote health, safety and well‐being	Collaborator	40	5,93	2,73	11	2
16. Uses family members as a source of information by verifying patient health history and medical, psychosocial, vocational, and financial condition	Communicator	41	5,90	2,50	10	1
44. Works with other professionals to support the development of and change in services (healthcare, educational and social) relevant to family‐centred care.	Leader	42	5,78	2,43	11	1
34. Establishes and maintains professional role boundaries with patients and family members	Collaborator	43	5,74	2,55	11	1
27. Acts as a contact liaison for patients and family members throughout all phases of care	Collaborator	44	5,74	2,73	11	1
45. Responds to health‐related issues or legal dilemmas in an ethical, moral, social and culturally congruent way in ways that empowers patients and family members	Health Advocate	45	5,72	2,81	11	1
38. Supports a culture that values diversity and promotes inclusion	Leader	46	5,71	2,36	11	1
4. Provides and reinforces education to patients and family members about diagnosis, treatment options, side effect management and posttreatment care	Nursing Expert	47	5,70	2,47	11	1
59. Assists and educates patients and family members to navigate the healthcare system by actively obtaining information, support and referral they need.	Health Advocate	48	5,68	2,54	11	1
71. Applies knowledge about ethics in encounters with family members regardless of age, sex or cultural background	Professional	49	5,62	2,38	10	1
14. Explains to and discusses with patients and family members why a particular treatment is inconsistent with the overall goals of care, using patients’ preferences as a rubric for why the treatment is not appropriate.	Communicator	50	5,52	2,50	11	2
70.Teaches and coaches family members on specific care skills	Scholar	51	5,52	2,51	11	1
39. Promotes patient‐ and family‐centred care as its own quality dimension that requires measurement and improvement	Leader	52	5,51	2,84	10	1
2. Applies knowledge of family dynamics and disease progression during interactions with patients and family members	Nursing Expert	53	5,49	2,39	11	1
67.Leads, or participates in, the evaluation of experiences of patients and family members	Scholar	54	5,48	2,07	11	2
11. Delivers bad news during a family meeting in a clear and compassionate manner	Communicator	55	5,38	2,07	11	1
60. Supports family members to identify, access and use resources relevant to their needs.	Health Advocate	56	5,38	2,15	10	1
63. Mentors others to incorporate patients and family members in the development of clinical care plans and goals.*	Scholar	57	5,28	2,23	11	1
72. Corroborates discussions and engage in problem solving overcoming complex issues regarding the delivery of family‐centred care	Professional	58	5,23	2,34	11	1
15. Encourages and facilitates communication about conflicts between patients and family members regarding goals of care.	Communicator	59	5,17	2,29	11	1
6. Assesses the family system and provides appropriate support to enable families to function as an adaptable network of caregivers.	Nursing Expert	60	5,17	2,48	11	1
61. Evaluates educational actions with patients and family members.	Scholar	61	4,90	2,31	11	1
52. Positively influence the health behaviours of patients and family members	Health Advocate	62	4,87	2,20	10	1
57. Recognizes that making surrogate decisions has a lasting emotional impact.	Health Advocate	63	4,81	2,38	10	1
68. Corroborates discussions with a broad focus among nurses, overcoming the eminently technical view and valuing ethics and human relations regarding family centred care.	Scholar	64	4,70	2,30	11	1
65. Has knowledge of one's own familial origins and experience and understands these can influence one's own behaviour, strengthening or stimulating behaviour.	Scholar	65	4,68	2,68	11	1
64. Develops a systematic method to assess the delivery of family centred care to decrease the risk of unwarranted variations in family‐centred care delivery	Scholar	66	4,58	2,57	11	1
51. Helps family members expand their vision of new opportunities and options	Health Advocate	67	4,52	2,37	10	1
62. Has knowledge of family systems and dynamics	Scholar	68	4,38	2,40	11	1
49. Performs an assessment and plans strategies to address socioeconomic factors influencing the ability of family members to care for the patient.	Health Advocate	69	3,96^‡^	2,53	11	1
55. Provides feedback on the reality of families’ life situations and how unhealthy choices may affect the lives of patients and family members.	Health Advocate	70	3,83^‡^	2,38	11	1
42. Utilizes technology that can help family members be familiar with community and other resources	Leader	71	3,67^‡^	2,24	8	1
58. Protects the family structure, which is under strain.	Health Advocate	72	3,26^c^	2,23	11	1

^a^ Significant Shapiro–Wilks test (median 9.0 IQR (4.5)),

^b^ Significant Shapiro–Wilks test (median 7.0 IQR (3.5)),

^c^ Significant Shapiro–Wilks test (median 3.0).

### 4.4 Quantitative post hoc analysis

In none of the subgroups the explained variance of two factors exceeded 29%, suggesting general lack of agreement on importance of competencies between and within our groups of professionals and countries. In Supplementary file S5, the results of the post‐hoc analysis of explained variance for subgroups are given.

### 4.5 Qualitative post hoc analysis: the five highest and lowest ranked competencies

Qualitative analysis revealed four themes from the highest ranked competencies and three themes from the lowest ranked competencies (Table 4). The four main themes from the highest ranked competencies are believed to: (a) be preconditions for FCC; (b) promote a partnership between nurses, patients and families; (c) be a basic element of nursing; and (d) represent a necessary positive attitude and strong beliefs of the added value of FCC. In the lowest ranked competencies, other themes were identified. The participants brought forward that these competencies: (a) are not a specific nursing competency; (b) demand a multidisciplinary approach; and (c) require that patients and families take own responsibility. An overview of the themes and supporting quotes is presented in Table 4.

**Table 4:** Themes and supporting quotes

Themes most important competencies	Quotes
Are preconditions for Family Centered Care.	‘…crucial to good support of patients and families.’
Promote a partnership between nurses, patients and families	‘…also facilitates trust between nurse, family and patient.’
Are a basic element of nursing.	‘…the foundation on which collaborative care can take place.’
Represent a necessary positive attitude and strong beliefs of the added value of Family Centered Care.	‘We must first acknowledge that family members can contribute significantly to care provision and need to be viewed as equal partners in decision making and care delivery.’

Themes least important competencies	Quotes
Not a specific nursing competency.	‘This is beyond the scope of nurses.’
Demand a multidisciplinary approach.	‘I think this would be better delivered as a multi‐disciplinary approach.’
Require that patients and families take own responsibility.	‘It seems to me the responsibilities of families themselves.’

## 5 DISCUSSION

### 5.1 Main findings

In our study, we found a set of competencies for FCC in the hospital setting. Using Q‐methodology we extracted two factors to aid in explaining different discourses on FCC among our participants, but the explained variance was too low for meaningful factor interpretation. Our results suggest that among our participants the concept of FCC evokes different ideas about needed competencies between different nursing professionals and these differences were also present in the separate groups of participants.

The highest ranked competencies are considered as a precondition for FCC. Among the competencies are the acknowledgment of family members as full partners in care, communicating with them in an honest and compassionate manner and supporting them in participating in clinical decision‐making. These competencies are mainly focused on one of the key components to facilitate FCC, namely the collaboration between family members and health care professionals to define care plans where family contexts are taken into account (Kokorelias et al., 2019), and highlights the fundamental values of being heard, respected, valued and supported by nurses (Frakking et al., 2020). This urges the importance of a strong relationship between nurses and family members to ensure consistency and continuity of care.

The participants emphasized the importance of competencies that represent a positive attitude and a strong belief in the added value of FCC. Some of these competencies are considered as a fundamental element of nursing care (e.g. adequate communication with families). Overall, the competencies that were ranked low, were considered beyond the scope of nursing (i.e. a required competency for social workers; or patient and families own responsibility) or were believed to require a multidisciplinary approach.

An adequate delivery of FCC by nurses is important as it confers many benefits to patients, but also to families and nurses themselves. A recent review of systematic reviews showed FCC interventions lower stress, anxiety and depression in families (Park et al., 2018). Furthermore, it enhances nurses’ motivation and thus their job satisfaction with less likelihood of burnout or intention to leave the nursing profession.

From the societal perspective there may be additional benefits as well. Worldwide the healthcare system is under immense pressure as the demand for care and costs will increase considerably in the upcoming years. (Williams et al., 2019) At the same time, organizations need to recruit and retain the necessary (nursing) staff to deliver a larger volume of exceedingly complex care. For example, at present, 1 in 7 people in the Netherlands work in healthcare. By 2040, when the aging of the Dutch population is at its peak, this will need to be 1 in 4 to keep up with the current demand for care. (Ministry of Health, 2018) This assignment seems to be impossible. It stands to reason that in the future, patients should seek more informal help in their own network. Despite the increasing demand of informal caregiving by families, exact numbers remain unclear (Bauer & Sousa‐Poza, 2015).

Hospital nurses are in a unique position to facilitate the core principles of family‐centred care including unbiased communication, collaboration in care and/or decision making and recognition of expertise. Many nurses agree that dealing with families of their patients is an important and a rewarding part of their professions, but this is not necessarily evident in their practice (Coyne et al., 2013). Lack of training of nurses and family members and communication issues are experienced as one of the main barriers for rooming‐in and the involvement of family members by nurses (van der Heijden et al., 2020). Consequently, capacity and capability of nurses to deliver FCC should be supported through initial and ongoing education, organizational support and explicated professional expectations so that nurses are well‐positioned to facilitate FCC in practice. Our set of competencies regarding FCC can inform the curriculum of initial nurse education and continuing education and is considered by the participants of our study as generalist competencies that each nurse working in a hospital should have.

In 2017, the International Family Nursing Association (IFNA) also published a set of competencies to promote family nursing practice. This set comprising five generalist and 33 core competencies is partly congruent with the competencies in our set. To give more insight in the similarities and differences between the two sets, we categorized and compared the competencies of both sets using the seven domains of the CanMEDs (see Supplementary file S6). There is considerable overlap in competencies in the domain of nursing expert, leader, health advocate, scholar and professional, however, our set contains more detailed competencies. The main differences between the two sets are found in the domain of communicator (e.g. more focus in our study on health literacy and psychosocial support to family members) and collaborator (e.g. more focus in our study on active family participation and assessing families’ preferred level of participation). We identified 23 competencies in our newly developed set, that we think are not present in, or described markedly different in the IFNA set (see Supplementary file S7). Most of these ranked low by our participants, but 3 ranked in the top 10. Furthermore, there is more attention for the cultural and ethical aspects of FCC in our set, although the cultural aspect of family nursing is addressed in competency 1.4 of the IFNA set.

### 5.2 Limitations of the study

Our study has several limitations. First, we did not include family members in our Q‐study. The reason for this is twofold. We wanted to explore competencies about nursing practice in those who would have to enact, teach or research them. Also, many of the competencies in our final set are complexly expressed and may not have been well understood by the public. This would negatively influence the accessibility of our study to a general audience of family members. Because we wanted to include as much information that was combined in each competency, we chose to not further adjust the wording for the competency. Second, several participants commented that they found it challenging to rank the competencies. Many found most of the competencies important (an average of 8 competencies where placed in the ‘least important category’ in the first step of the Q‐sort process, data not shown). Given the fact the Q‐set is based on a review of the literature looking for competencies for FCC, this was to be expected. Keeping an overview of the large number of competencies also proved difficult for some. Several participants stated that some competencies are rather generic statements that they felt are not specific to FCC. Nevertheless, we think that using Q‐sorting was the best option to rank so many competencies in a meaningful way, without having the time to do a full pairwise comparison. Third, because the analyses resulted in two factors with a low explained variance, we decided not to proceed to the phase of interpretation. As a result, meaningful labelling and a detailed description of the factors are lacking in this study. We substantiate this method, because we do not want to give the impression that our analyses resulted in a set of synthesized, shared perspectives, which is not supported by the data. Instead, we present an extensive table of items with the relevant ranking information. Additionally, we explored the low explained variance, but also in subgroups we found similar results. Lastly, we acknowledge the subjective nature of the comparison between our newly developed set and the IFNA set. The difference in descriptions made direct comparison difficult.

## 6 CONCLUSION

To the best of our knowledge, this is the first study that rigorously searched the published literature of nursing competencies for FFC and analysed the importance professionals attach to them. Although Family nursing and Family‐centred care can be considered different (Bell, 2013), our list of competencies overlaps those issued in IFNA position statement and can be seen as a (first) validation of their set for the hospital setting. We feel our comparison of the two sets of competencies can provide ground for further discussion on breadth and description of competencies in both sets. Developing a set of competencies that is both practical in size and detailed enough is a challenge for future research. More studies regarding this topic are needed to enlarge the evidence base on competencies for FCC, where special emphasis should be placed on geographical and cultural differences and the effect of their teaching and implementation on patient and family outcomes. Furthermore, future research on this topic should be aimed at including the full spectrum of family members. This means that the description of competencies should be looked at as they might be difficult to comprehend for people with lower literacy levels. We also think future research should include vocationally educated registered nurses and licensed practical nurses. We feel that the results of our study serve as a good starting point for such research. Using Q‐sorting to rank a rather large set of competencies proved to be both useful and challenging. In our initial study protocol, an ordinary high‐low ranking was envisioned. Testing this using an online sorting tool proved to be impossible given the large number of competencies. The way Q‐sorting divides the sorting process in two steps and using grouping made it possible to rank the competencies. Although the use of the HTMLQ software served us in our needs, we feel the development of new and easily configurable software in the public domain would benefit this type of research greatly. Reducing the number of competencies will very likely improve the usability of the set, both in research and in practice. Replication studies could guide this process, by choosing the most important competencies. Our set of competencies can be used to guide education and evaluate practicing nurses in hospitals. Based on our findings we think any implementation of FCC into practice should consider different views on FCC that might exist among stakeholders.

## CONFLICT OF INTERESTS

None.

## AUTHOR CONTRIBUTIONS

**Bram Hengeveld:** Conceived the idea for and planned the study, Conceived and designed the analysis of each phase, Built the HTMLQ‐website, Collected the data, Contributed data and analysis tools, Performed the analysis, Wrote the paper, Gave approval for submission. **Jolanda M. Maaskant:** Conceived and designed the analysis of phase 1, 2 and 3, Performed the qualitative analysis of competencies, Wrote the paper, Gave approval for submission. **Robert Lindeboom:** Conceived and designed the analysis of each phase, Performed the analysis, Wrote the paper, Gave approval for submission. **Andrea Marshall:** Conceived the idea for and planned the study, Contributed data and analysis tools, Wrote the paper, Gave approval for submission. **Hester Vermeulen:** Conceived the idea for and planned the study, Wrote the paper, Gave approval for submission. **Anne Eskes:** Conceived the idea for and planned the study, Conceived and designed the analysis of phase 1, 2 and 3, Performed the qualitative analysis of competencies, Wrote the paper, Gave approval for submission.

### Peer Review

The peer review history for this article is available at https://publons.com/publon/10.1111/jan.14719.

## Supporting information

Supplementary Material
