# Supplementary material for: Nursing competencies for family‐centred care in the hospital setting: A multinational Q‐methodology study
Source: J Adv Nurs. 2020 Dec 13;77(4):1783–99. doi: 10.1111/jan.14719 (PMC8048472; doi:10.1111/jan.14719)
Supplement: Supplementary file 1 — Supplementary Material [file JAN-77-1783-s001.pdf]

SUPPLEMENTARY MATERIAL

Contents

	Page
Supplementary file 1 - PubMed Search string	2
Supplementary file 2 - An example of the online Q-sort	3
Supplementary file 3 - List of included publications in integrative review	4
Supplementary file 4 - List of competencies grouped according to CanMeds roles	7
Supplementary file 5 - Post-hoc analysis of explained variance for subgroups	11
Supplementary file 6 - Comparison of IFNA generalist competencies versus the newly developed set	12
Nursing expert	12
Communicator	14
Collaborator	19
Leader	21
Health Advocate	23
Scholar	26
Professional	28
Supplementary file 7 – Unique competencies in our developed set	29

Supplementary file 1 – PubMed Search string

PubMed Search string	
1	"Nursing"[MeSH Terms] OR “Nursing Staff”[MeSH Terms] OR "Family Nurse Practitioners"[MeSH Terms] OR "Nurse's Role"[MeSH Terms] OR nursing[All Fields] OR nurse[All Fields] OR nurses[All Fields] OR nurs*[All Fields]
2	"clinical competence"[MeSH Terms] OR "clinical competence"[tiab] OR Competencies[tiab] OR Competenc*[tiab] OR Competency[tiab] OR Competence[tiab]
3	("family"[MeSH Terms] OR "family"[All Fields]) AND (centred[All Fields] OR centered[ALL Fields] OR delivered[All Fields] OR integrated[All Fields]) AND (care[All Fields])
4	("family"[MeSH Terms] OR "family"[All Fields]) AND (centered[All Fields] OR centered[ALL Fields] OR delivered[All Fields] OR integrated[All Fields]) AND ("intensive care units, neonatal"[MeSH Terms] OR ("intensive"[All Fields] AND "care"[All Fields] AND "units"[All Fields] AND "neonatal"[All Fields]) OR "neonatal intensive care units"[All Fields] OR "nicu"[All Fields])
5	("family"[MeSH Terms] OR "family"[All Fields]) AND (centered[All Fields] OR centered[ALL Fields] OR delivered[All Fields] OR "integrated"[All Fields]) AND ("intensive care units"[MeSH Terms] OR ("intensive"[All Fields] AND "care"[All Fields] AND "units"[All Fields]) OR "intensive care units"[All Fields] OR "icu"[All Fields])
6	"case management"[MeSH Terms] OR casemanagement[All Fields] OR "case management"[All Fields]
7	“caregivers”[MeSH Terms] OR caregiver*[All Fields] OR family caregiver*[All Fields] OR "Family centered care"[All Fields] OR "Family centred care"[All Fields] OR "Family centered nicu"[All Fields] OR "Family centred nicu"[All Fields] OR "Family centered icu"[All Fields] OR "Family centred icu"[All Fields] OR “Family delivered care”[All Fields] OR "Family integrated care"[All Fields] OR “Family care”[All Fields] OR “Family participation”[All Fields] OR "Family engagement"[All Fields] OR “Family involvement”[All Fields] OR “Family collaboration”[All Fields] OR "Informal caregiver”[All Fields] OR “family Nursing”[Mesh] OR “family Nursing”[All Fields] OR “Family activation”[All Fields] OR “Caregiver activation”[All Fields]
8	#1 AND #2 AND (#3 OR #4 OR #5 OR #6 OR #7)

Supplementary file 2 – An example of the online Q-sort

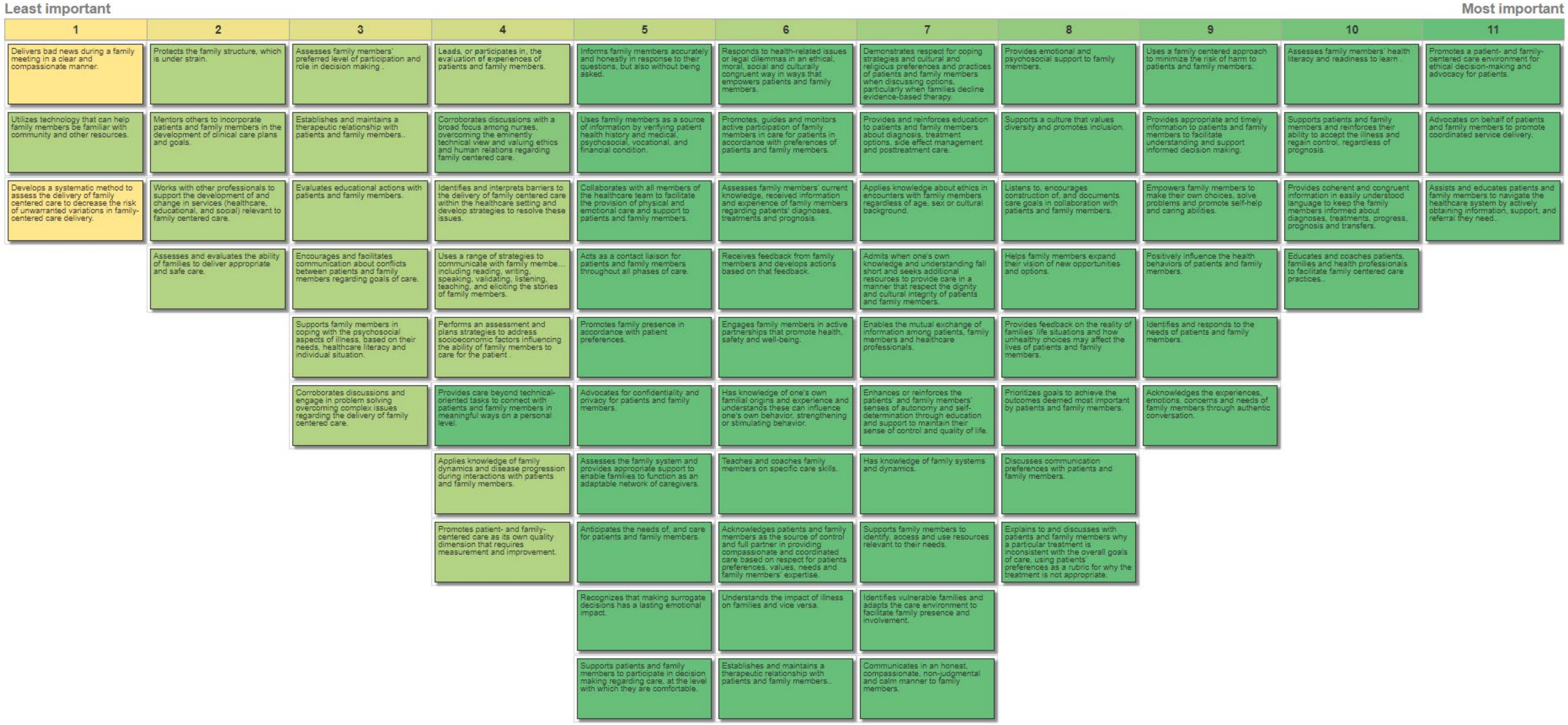

Supplementary file 2 - Figure Example of one of the online Q-sorts. Yellow cards were placed in the 'least important' category during step 1 of the sorting process, light green cards in the 'neutral' category and dark green cards in the 'most important' category

Supplementary file 3 – List of included publications in integrative review

List of included publications in integrative review

Alfieri, E., Alebbi, A., Bedini, M. G., Boni, L., & Foa, C. (2017). Mapping the nursing competences in neonatology: a qualitative research. *Acta Biomed*, 88(3), 51-58.

Alfieri, E., Ferrini, A. C., Gianfrancesco, F., Lise, G., Messana, G., Tirelli, L., . . . Sarli, L. (2017). The mapping competences of the nurse Case/Care Manager in the context of Intensive Care. *Acta Biomed*, 88(1), 69-75.

Arias Murcia, S. E., & Lopez, L. (2016). The experience of nurses in care for culturally diverse families: A qualitative meta-synthesis. *Revista Latino-Americana de Enfermagem* (RLAE), 24, 1-11.

Barton, J. A., & Brown, N. J. (1995). Home visitation to migrant farm worker families: an application of Zerwekh's family caregiving model for public health nursing. *Holist Nurs Pract,* 9(4), 34-40.

Burke, K. G., Johnson, T., Sites, C., & Barnsteiner, J. (2017). CE: Original Research: Creating an Evidence-Based Progression for Clinical Advancement Programs. *Am J Nurs*, 117(5), 22-35.

Collins, A. M., & Reinke, E. (1997). Use of a family caregiving model to articulate the role of the public health nurse in infant mental health promotion. *Issues Compr Pediatr Nurs*, 20(4), 207-216.

Cook, S., Fillion, L., Fitch, M., Veillette, A.-M., Matheson, T., Aubin, M., . . . Rainville, F. (2013). Core areas of practice and associated competencies for nurses working as professional cancer navigators. *Canadian Oncology Nursing Journal*, 23(1), 44-52.

Cypress, B. S. (2013). Using the synergy model of patient care in understanding the lived emergency department experiences of patients, family members and their nurses during critical illness: a phenomenological study. *Dimens Crit Care Nurs,* 32(6), 310-321.

Dale, C., & Storey, L. (2004). Focus. High, medium, and low security care: does the type of care make any difference to the role of the forensic mental health nurse? *NT Research*, 9(3), 168-184.

Duhamel, F., & Dupuis, F. (2004). Guaranteed returns: investing in conversations with families of patients with cancer. *Clin J Oncol Nurs*, 8(1), 68-71.

Engler, A. J., Cusson, R. M., Brockett, R. T., Cannon-Heinrich, C., Goldberg, M. A., West, M. G., & Petow, W. (2004). Neonatal staff and advanced practice nurses' perceptions of bereavement/end-of-life care of families of critically ill and/or dying infants. *American Journal of Critical Care*, 13(6), 489-498.

Hart, P. L., & Mareno, N. (2014). Cultural challenges and barriers through the voices of nurses. *Journal of Clinical Nursing*, 23(15), 2223-2233.

Haruta, J., Yoshida, K., Goto, M., Yoshimoto, H., Ichikawa, S., Mori, Y., . . . Otsuka, M. (2018). Development of an interprofessional competency framework for collaborative practice in Japan. J Interprof Care, 1-8.

Hibberd, P. (2011). What is the meaning of family-centred Admiral Nursing for carers? , University of Northumbria at Newcastle (United Kingdom), Ann Arbor.

Holloway, R. G., Arnold, R. M., Creutzfeldt, C. J., Lewis, E. F., Lutz, B. J., McCann, R. M., . . . Zorowitz, R. D. (2014). Palliative and end-of-life care in stroke: a statement for healthcare professionals from the American Heart Association/American Stroke Association. *Stroke*, 45(6), 1887-1916.

Hull, M. M. (1991). Hospice nurses. Caring support for caregiving families. *Cancer Nurs*, 14(2), 63-70.

Kiljunen, O., Kankkunen, P., Partanen, P., & Valimaki, T. (2017). Family members' expectations regarding nurses' competence in care homes: a qualitative interview study. *Scand J Caring Sci*.

Lewis, L. (2008). Family caregiving. Discussion and recommendations. *Am J Nurs*, 108(9), 83-87.

Lowey, S. E. (2008). Letting go before a death: a concept analysis. *J Adv Nurs*, 63(2), 208-215.

Lynn, M. (2008). Lessons Learned While Studying Quality Home Health Care. *Southern Online Journal of Nursing Research*, 8(4), 10-10.

McCallum, M., Carver, J., Dupere, D., Ganong, S., Henderson, J. D., McKim, A., . . . Jewers, H. (2018). Developing a Palliative Care Competency Framework for Health Professionals and Volunteers: The Nova Scotian Experience. *J Palliat Med,* 21(7), 947-955.

McEnroe, L. E. (1996). Role of the oncology nurse in home care: family-centered practice. *Semin Oncol Nurs*, 12(3), 188-192.

McMullen, L., Banman, T., DeGroot, J. M., Scott, S., Srdanovic, D., & Mackey, H. (2016). Providing Novice Navigators With a GPS for Role Development: Oncology Nurse Navigator Competency Project. *Clin J Oncol Nurs*, 20(1), 33-38.

Mendes, M. A. (2013). Parents' descriptions of ideal home nursing care for their technology-dependent children. *Pediatr Nurs*, 39(2), 91-96.

Moe, K., Valen-Sendstad Skisland , A., & Söderhamn, U. (2017). Encountering parents of a sick newborn child. *Norwegian Journal of Clinical Nursing / Sykepleien Forskning*, 1-11.

Morgan, D. G., Kosteniuk, J. G., O'Connell, M. E., Dal Bello-Haas, V., Stewart, N. J., & Karunanayake, C. (2016). Dementia-Related Work Activities of Home Care Nurses and Aides: Frequency, Perceived Competence, and Continuing Education Priorities. *Educational Gerontology*, 42(2), 120-135.

Mularski, R. A., Bascom, P., & Osborne, M. L. (2001). Educational agendas for interdisciplinary end-of-life curricula. *Crit Care Med*, 29(2), N16-23.

Nailon, R. E. R. (2004). *Expertise in the care of Latinos: an interpretive study of culturally congruent nursing practices in the emergency department*. Oregon Health & Science University, Portland (OR).

O'Connell, E., & Landers, M. (2008). The importance of critical care nurses' caring behaviours as perceived by nurses and relatives. *Intensive Crit Care Nurs*, 24(6), 349-358.

Oliveira, R. G., & Marcon, S. S. (2007). The opinion of nurses regarding the work they perform with families in the family health program. *Rev Lat Am Enfermagem*, 15(3), 431-438.

Renehan, E., Goeman, D., & Koch, S. (2017). Development of an optimised key worker framework for people with dementia, their family and caring unit living in the community. BMC *Health Serv Res*, 17(1), 501.

Roets, L., Rowe-Rowe, N., & Nel, R. (2012). Family-centred care in the paediatric intensive care unit. *J Nurs Manag*, 20(5), 624-630.

Saylor, J. L., Schell, K. A., Mendell, M. F., & Graber, J. S. (2015). Development of a Clinical Competency Checklist for Care of Patients Experiencing Substance Withdrawal Delirium or Delirium: Use of a Delphi Technique and Expert Panel. *J Psychosoc Nurs Ment Health Serv*, 53(6), 29-36.

Selman, L. E., Brighton, L. J., Sinclair, S., Karvinen, I., Egan, R., Speck, P., . . . Hope, J. (2018). Patients' and caregivers' needs, experiences, preferences and research priorities in spiritual care: A focus group study across nine countries. *Palliat Med*, 32(1), 216-230.

Skene, C., Gerrish, K., Price, F., Pilling, E., Bayliss, P., & Gillespie, S. (2018). Developing family-centred care in a neonatal intensive care unit: An action research study. *Intensive Crit Care Nurs.*

Stokes, D. (2016). Empowering Children with Autism Spectrum Disorder and Their Families within the Healthcare Environment. *Pediatr Nurs*, 42(5), 254-255.

Suemi Kawata, L., Martins Mishima, S., Quaglio Chirelli, M., JosÃ© Bistafa Pereira, M., Matumoto, S., & Fortuna, C. M. (2013). The performances of the nurse in family health – building competence for care. *Texto & Contexto Enfermagem,* 22(4), 961-970.

Swartwout, E., Drenkard, K., McGuinn, K., Grant, S., & El-Zein, A. (2016). Patient and Family Engagement Summit: Needed Changes in Clinical Practice. *J Nurs Adm*, 46(3), S11-18.

Tahan, H. M., Watson, A. C., & Sminkey, P. V. (2015). What Case Managers Should Know About Their Roles and Functions: A National Study From the Commission for Case Manager Certification: Part 1. *Prof Case Manag*, 20(6), 271-296

Traynor, V., Inoue, K., & Crookes, P. (2011). Literature review: understanding nursing competence in dementia care. *J Clin Nurs*, 20(13), 1948-1960.

Valéria Marli, L., Milene Pires de Moraes, V., & Thalita Cristine Ramirez, D. (2018). Competencies for educational actions of Family Health Strategy nurses. *Revista Brasileira de Enfermagem,* 71(3), 1072-1078.

White, K. R., Coyne, P. J., & White, S. G. (2012). Are hospice and palliative nurses adequately prepared for end-of-life care? *Journal of Hospice & Palliative Nursing*, 14(2), 133-140.

Woodring, B. C., & Pridham, K. F. (1998). Standards and Guidelines for Pre-Licensure and Early Professional Education for the Nursing Care of Children and Their Families. [Revised].

Supplementary file 4– List of competencies grouped according to CanMeds roles

List of competencies grouped according to CanMeds role	
**Competency (the nurse…)**	**CanMeds Role**
1. Identifies and responds to the needs of patients and family members.	Nursing Expert
2. Applies knowledge of family dynamics and disease progression during interactions with patients and family members	Nursing Expert
3. Anticipates the needs of, and care for patients and family members	Nursing Expert
4. Provides and reinforces education to patients and family members about diagnosis, treatment options, side effect management and posttreatment care	Nursing Expert
5. Uses a family centered approach to minimize the risk of harm to patients and family members	Nursing Expert
6. Assesses the family system and provides appropriate support to enable families to function as an adaptable network of caregivers.	Nursing Expert
7. Assesses and evaluates the ability of families to deliver appropriate and safe care	Nursing Expert
8. Supports family members in coping with the psychosocial aspects of illness, based on their needs, healthcare literacy and individual situation	Communicator
9. Acknowledges the experiences, emotions, concerns and needs of family members through authentic conversation	Communicator
10. Provides emotional and psychosocial support to family members	Communicator
11. Delivers bad news during a family meeting in a clear and compassionate manner	Communicator
12. Listens to, encourages construction of, and documents care goals in collaboration with patients and family members	Communicator
13. Prioritizes goals to achieve the outcomes deemed most important by patients and family members	Communicator
14. Explains to and discusses with patients and family members why a particular treatment is inconsistent with the overall goals of care, using patients' preferences as a rubric for why the treatment is not appropriate.	Communicator
15. Encourages and facilitates communication about conflicts between patients and family members regarding goals of care.	Communicator
16. Uses family members as a source of information by verifying patient health history and medical, psychosocial, vocational, and financial condition	Communicator
17. Assesses family members' health literacy and readiness to learn.	Communicator
18. Assesses family members' current knowledge, received information and experience of family members regarding patients' diagnoses, treatments and prognosis.	Communicator

List of competencies grouped according to CanMeds role	
**Competency (the nurse…)**	**CanMeds Role**
19. Uses a range of strategies to communicate with family members, including reading, writing, speaking, validating, listening, teaching, and eliciting the stories of family members	Communicator
20. Provides coherent and congruent information in easily understood language to keep the family members informed about diagnoses, treatments, progress, prognosis and transfers.	Communicator
21. Communicates in an honest, compassionate, non-judgmental and calm manner to family members	Communicator
22. Provides appropriate and timely information to patients and family members to facilitate understanding and support informed decision making	Communicator
23. Discusses communication preferences with patients and family members	Communicator
24. Provides care beyond technical-oriented tasks to connect with patients and family members in meaningful ways on a personal level.	Communicator
25. Demonstrates respect for coping strategies and cultural and religious preferences and practices of patients and family members when discussing options, particularly when families decline evidence-based therapy	Communicator
26. Establishes and maintains a therapeutic relationship with patients and family members.	Communicator
27. Acts as a contact liaison for patients and family members throughout all phases of care	Collaborator
28. Acknowledges patients and family members as the source of control and full partner in providing compassionate and coordinated care based on respect for patients' preferences, values, needs and family members' expertise.	Collaborator
29. Assesses family members' preferred level of participation and role in decision making	Collaborator
30. Engages family members in active partnerships that promote health, safety and well-being	Collaborator
31. Promotes family presence in accordance with patient preferences	Collaborator
32. Promotes, guides and monitors active participation of family members in care for patients in accordance with preferences of patients and family members.	Collaborator
33. Collaborates with all members of the healthcare team to facilitate the provision of physical and emotional care and support to patients and family members	Collaborator
34. Establishes and maintains professional role boundaries with patients and family members	Collaborator
35. Supports patients and family members to participate in decision making regarding care, at the level with which they are comfortable.	Collaborator

List of competencies grouped according to CanMeds role	
**Competency (the nurse…)**	**CanMeds Role**
36. Enables the mutual exchange of information among patients, family members and healthcare professionals	Collaborator
37. Informs family members accurately and honestly in response to their questions, but also without being asked.	Collaborator
38. Supports a culture that values diversity and promotes inclusion	Leader
39. Promotes patient- and family-centered care as its own quality dimension that requires measurement and improvement	Leader
40. Identifies and interprets barriers to the delivery of family centered care within the healthcare setting and develop strategies to resolve these issues	Leader
41. Educates and coaches patients, families and health professionals to facilitate family centered care practices.	Leader
42. Utilizes technology that can help family members be familiar with community and other resources	Leader
43. Promotes a patient- and family-centered care environment for ethical decision-making and advocacy for patients	Leader
44. Works with other professionals to support the development of and change in services (healthcare, educational, and social) relevant to family centered care.	Leader
45. Responds to health-related issues or legal dilemmas in an ethical, moral, social and culturally congruent way in ways that empowers patients and family members	Health Advocate
46. Identifies vulnerable families and adapts the care environment to facilitate family presence and involvement	Health Advocate
47. Advocates on behalf of patients and family members to promote coordinated service delivery	Health Advocate
48. Advocates for confidentiality and privacy for patients and family members	Health Advocate
49. Performs an assessment and plans strategies to address socioeconomic factors influencing the ability of family members to care for the patient.	Health Advocate
50. Understands the impact of illness on families and vice versa	Health Advocate
51. Helps family members expand their vision of new opportunities and options	Health Advocate
52. Positively influence the health behaviors of patients and family members	Health Advocate
53. Empowers family members to make their own choices, solve problems and promote self-help and caring abilities	Health Advocate
54. Supports patients and family members and reinforces their ability to accept the illness and regain control, regardless of prognosis	Health Advocate
55. Provides feedback on the reality of families' life situations and how unhealthy choices may affect the lives of patients and family members.	Health Advocate

List of competencies grouped according to CanMeds role

**Competency (the nurse…)**	**CanMeds Role**
56. Enhances or reinforces the patients' and family members' senses of autonomy and self-determination through education and support to maintain their sense of control and quality of life	Health Advocate
57. Recognizes that making surrogate decisions has a lasting emotional impact.	Health Advocate
58. Protects the family structure, which is under strain.	Health Advocate
59. Assists and educates patients and family members to navigate the healthcare system by actively obtaining information, support, and referral they need.	Health Advocate
60. Supports family members to identify, access and use resources relevant to their needs.	Health Advocate
61. Evaluates educational actions with patients and family members.	Scholar
62. Has knowledge of family systems and dynamics	Scholar
63. Mentors others to incorporate patients and family members in the development of clinical care plans and goals.	Scholar
64. Develops a systematic method to assess the delivery of family centered care to decrease the risk of unwarranted variations in family-centered care delivery	Scholar
65. Has knowledge of one's own familial origins and experience and understands these can influence one's own behavior, strengthening or stimulating behavior.	Scholar
66. Admits when one's own knowledge and understanding fall short and seeks additional resources to provide care in a manner that respect the dignity and cultural integrity of patients and family members.	Scholar
67.Leads, or participates in, the evaluation of experiences of patients and family members	Scholar
68. Corroborates discussions with a broad focus among nurses, overcoming the eminently technical view and valuing ethics and human relations regarding family centered care.	Scholar
69. Receives feedback from family members and develops actions based on that feedback.	Scholar
70.Teaches and coaches family members on specific care skills	Scholar
71. Applies knowledge about ethics in encounters with family members regardless of age, sex or cultural background	Professional
72. Corroborates discussions and engage in problem solving overcoming complex issues regarding the delivery of family centered care	Professional

Health Advocate = Health Advocate.

Supplementary file 5 - Post-hoc analysis of explained variance for subgroups

	N	Total explained variance in first two factors (%)
**By country**		
Australians only	35	26
Dutch only	34	26
**By professional group 1**		
Frontline healthcare professionals (i.e. nurses only)*	20	29
Non-frontline healthcare professionals*	49	24
**By professional group 2**		
Nurses only**	18	29
Lecturers only	15	23
Researchers only	23	26
Policy makers only	13	26
**Leave-one-out**		
Lectures, researchers & policy makers	51	24
Nurses, researchers & policy makers	54	24
Nurses, lecturers & policy makers	46	26
Nurses, researchers & lecturers	56	24

This is an exploratory analysis per subgroup. The assumptions to use factor analysis as defined in the method section are not fulfilled.

* Three professionals combined frontline healthcare work with non-frontline healthcare work.

** Main professional group

Supplementary file 6 – Comparison of IFNA generalist competencies versus the newly developed set

Approval of reproduction of the IFNA set is given by Ms. D. Zaparoni. Microsoft Visio files are available on request.

Nursing expert

Comparison of competencies in our set belonging to the CanMeds role of nursing expert (left) and the complete IFNA generalist set (right). We found no connection with domains 3 and 4 of the IFNA set

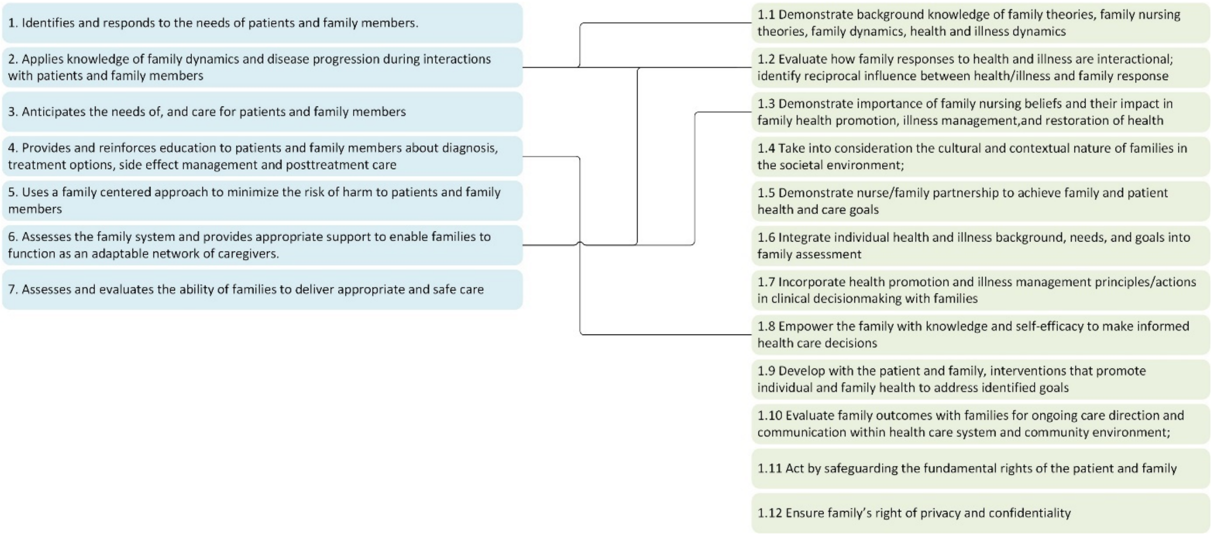

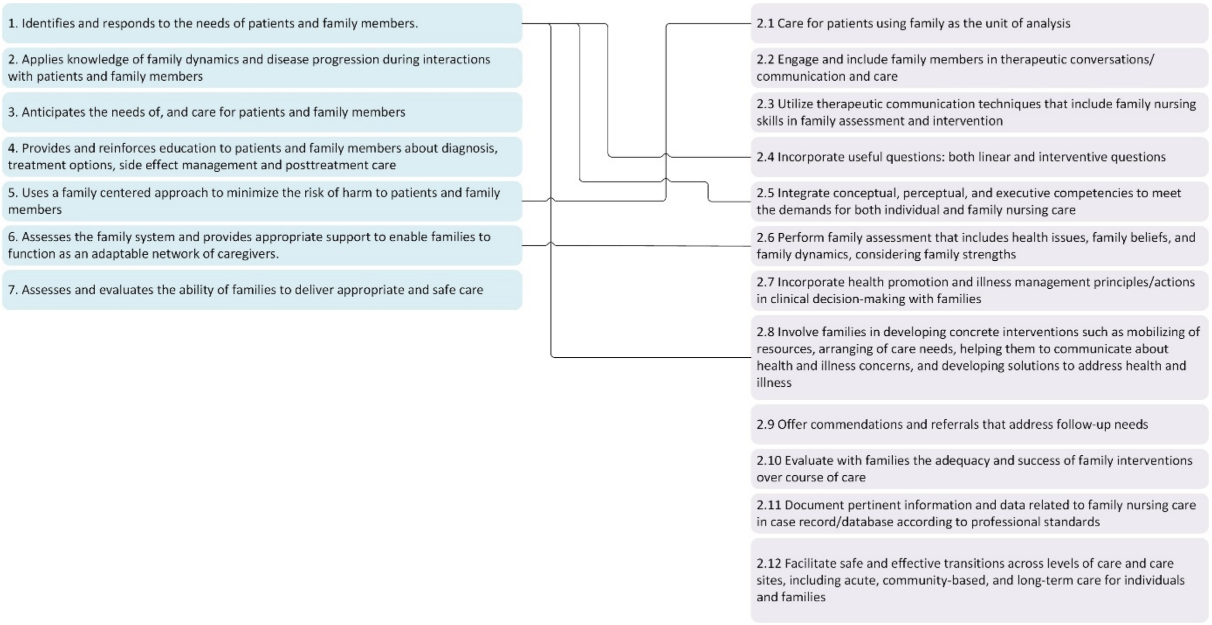

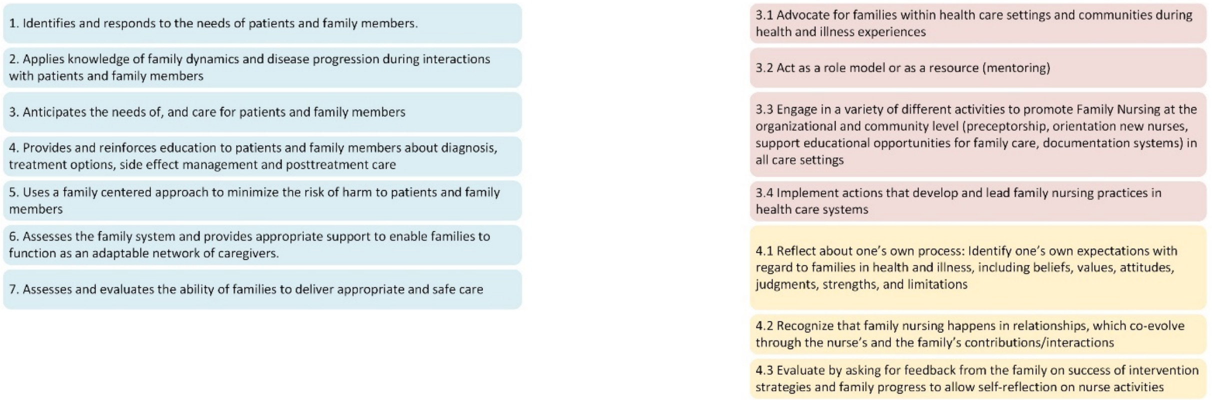

1. Identifies and responds to the needs of patients and family members.

2. Applies knowledge of family dynamics and disease progression during interactions with patients and family members

3. Anticipates the needs of, and care for patients and family members

4. Provides and reinforces education to patients and family members about diagnosis, treatment options, side effect management and posttreatment care

5. Uses a family centered approach to minimize the risk of harm to patients and family members

- 5.1 Apply a nurse-family practice model that recognizes the significance of family and societal systems in health
- 5.2 Utilize research and practice based evidence to support family assessment, interventions, and care with families

6. Assesses the family system and provides appropriate support to enable families to function as an adaptable network of caregivers.

7. Assesses and evaluates the ability of families to deliver appropriate and safe care

Communicator

Comparison of competencies in our set belonging to the CanMeds role of Communicator (left) and the complete IFNA generalist set (right). We found no connection with domain 3 of the IFNA set.

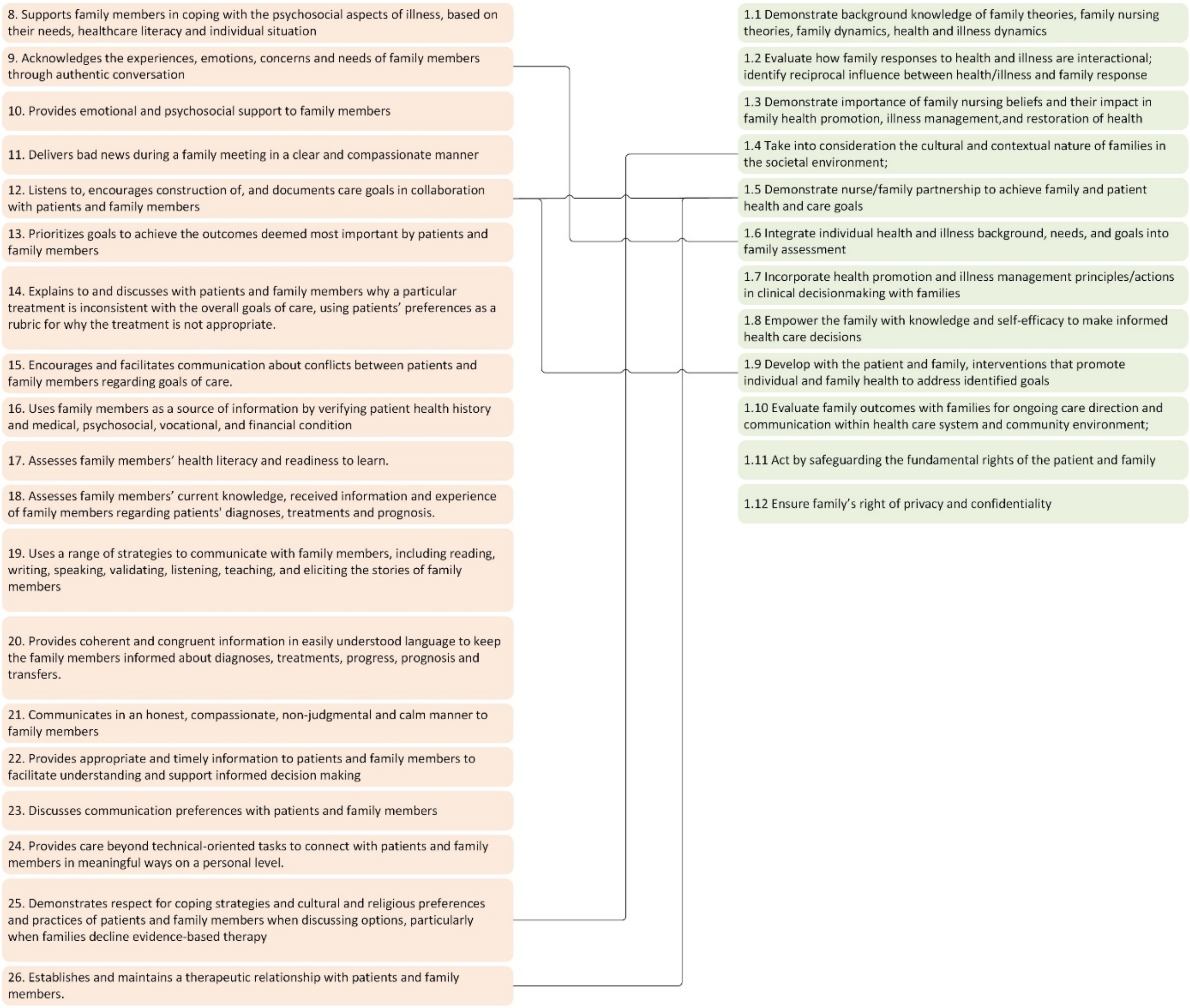

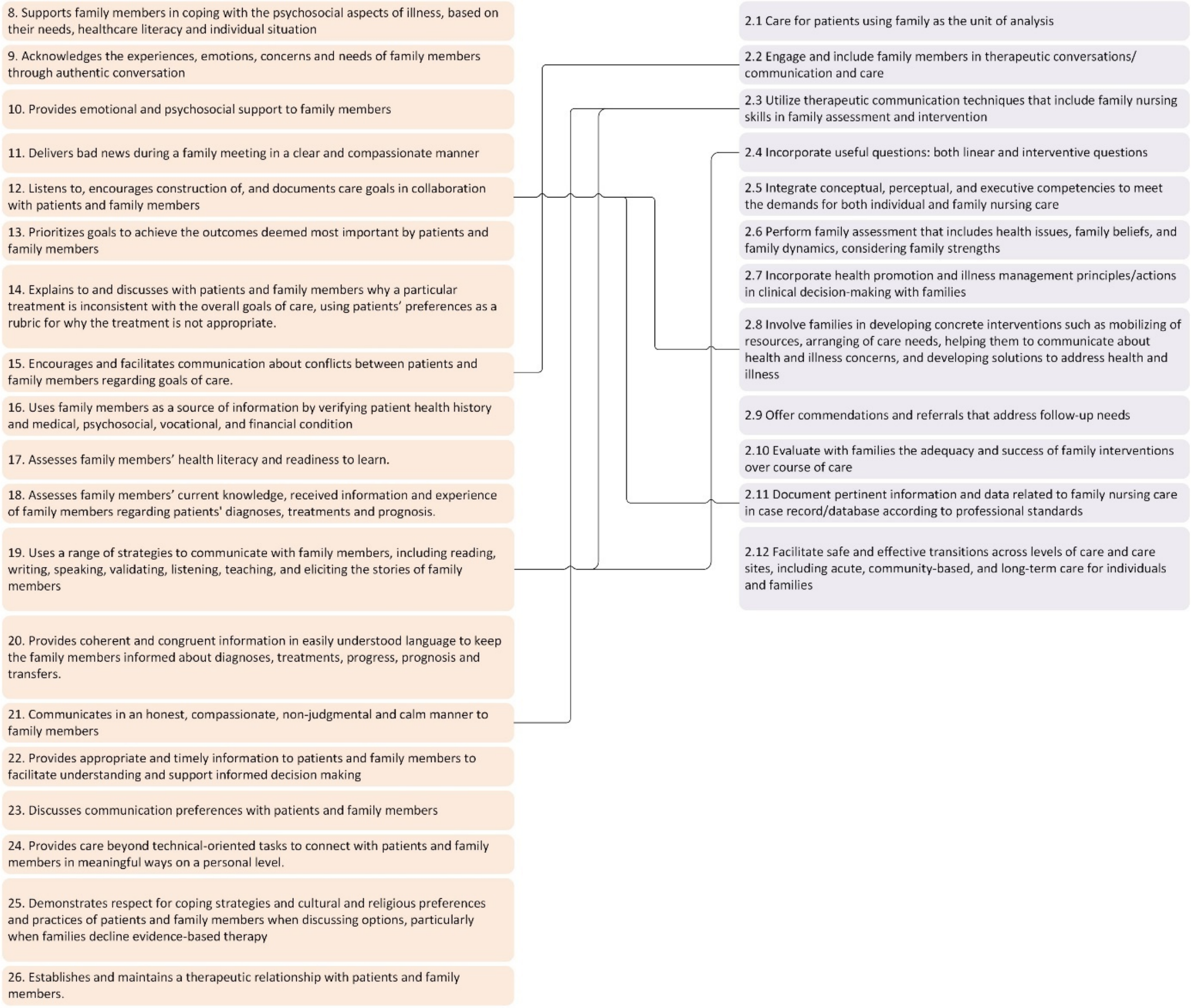
8. Supports family members in coping with the psychosocial aspects of illness, based on their needs, healthcare literacy and individual situation
9. Acknowledges the experiences, emotions, concerns and needs of family members through authentic conversation
10. Provides emotional and psychosocial support to family members
11. Delivers bad news during a family meeting in a clear and compassionate manner
12. Listens to, encourages construction of, and documents care goals in collaboration with patients and family members
13. Prioritizes goals to achieve the outcomes deemed most important by patients and family members
14. Explains to and discusses with patients and family members why a particular treatment is inconsistent with the overall goals of care, using patients' preferences as a rubric for why the treatment is not appropriate.
15. Encourages and facilitates communication about conflicts between patients and family members regarding goals of care.
16. Uses family members as a source of information by verifying patient health history and medical, psychosocial, vocational, and financial condition
17. Assesses family members' health literacy and readiness to learn.
18. Assesses family members' current knowledge, received information and experience of family members regarding patients' diagnoses, treatments and prognosis.
19. Uses a range of strategies to communicate with family members, including reading, writing, speaking, validating, listening, teaching, and eliciting the stories of family members
20. Provides coherent and congruent information in easily understood language to keep the family members informed about diagnoses, treatments, progress, prognosis and transfers.
21. Communicates in an honest, compassionate, non-judgmental and calm manner to family members
22. Provides appropriate and timely information to patients and family members to facilitate understanding and support informed decision making
23. Discusses communication preferences with patients and family members
24. Provides care beyond technical-oriented tasks to connect with patients and family members in meaningful ways on a personal level.
25. Demonstrates respect for coping strategies and cultural and religious preferences and practices of patients and family members when discussing options, particularly when families decline evidence-based therapy
26. Establishes and maintains a therapeutic relationship with patients and family members.
2.1 Care for patients using family as the unit of analysis
2.2 Engage and include family members in therapeutic conversations/ communication and care
2.3 Utilize therapeutic communication techniques that include family nursing skills in family assessment and intervention
2.4 Incorporate useful questions: both linear and interventive questions
2.5 Integrate conceptual, perceptual, and executive competencies to meet the demands for both individual and family nursing care
2.6 Perform family assessment that includes health issues, family beliefs, and family dynamics, considering family strengths
2.7 Incorporate health promotion and illness management principles/actions in clinical decision-making with families
2.8 Involve families in developing concrete interventions such as mobilizing of resources, arranging of care needs, helping them to communicate about health and illness concerns, and developing solutions to address health and illness
2.9 Offer commendations and referrals that address follow-up needs
2.10 Evaluate with families the adequacy and success of family interventions over course of care
2.11 Document pertinent information and data related to family nursing care in case record/database according to professional standards
2.12 Facilitate safe and effective transitions across levels of care and care sites, including acute, community-based, and long-term care for individuals and families

8. Supports family members in coping with the psychosocial aspects of illness, based on their needs, healthcare literacy and individual situation

9. Acknowledges the experiences, emotions, concerns and needs of family members through authentic conversation

10. Provides emotional and psychosocial support to family members

11. Delivers bad news during a family meeting in a clear and compassionate manner

12. Listens to, encourages construction of, and documents care goals in collaboration with patients and family members

13. Prioritizes goals to achieve the outcomes deemed most important by patients and family members

14. Explains to and discusses with patients and family members why a particular treatment is inconsistent with the overall goals of care, using patients' preferences as a rubric for why the treatment is not appropriate.

15. Encourages and facilitates communication about conflicts between patients and family members regarding goals of care.

16. Uses family members as a source of information by verifying patient health history and medical, psychosocial, vocational, and financial condition

17. Assesses family members' health literacy and readiness to learn.

18. Assesses family members' current knowledge, received information and experience of family members regarding patients' diagnoses, treatments and prognosis.

19. Uses a range of strategies to communicate with family members, including reading, writing, speaking, validating, listening, teaching, and eliciting the stories of family members

20. Provides coherent and congruent information in easily understood language to keep the family members informed about diagnoses, treatments, progress, prognosis and transfers.

21. Communicates in an honest, compassionate, non-judgmental and calm manner to family members

22. Provides appropriate and timely information to patients and family members to facilitate understanding and support informed decision making

23. Discusses communication preferences with patients and family members

24. Provides care beyond technical-oriented tasks to connect with patients and family members in meaningful ways on a personal level.

25. Demonstrates respect for coping strategies and cultural and religious preferences and practices of patients and family members when discussing options, particularly when families decline evidence-based therapy

26. Establishes and maintains a therapeutic relationship with patients and family members.

3.1 Advocate for families within health care settings and communities during health and illness experiences

3.2 Act as a role model or as a resource (mentoring)

3.3 Engage in a variety of different activities to promote Family Nursing at the organizational and community level (preceptorship, orientation new nurses, support educational opportunities for family care, documentation systems) in all care settings

3.4 Implement actions that develop and lead family nursing practices in health care systems

8. Supports family members in coping with the psychosocial aspects of illness, based on their needs, healthcare literacy and individual situation

9. Acknowledges the experiences, emotions, concerns and needs of family members through authentic conversation

10. Provides emotional and psychosocial support to family members

11. Delivers bad news during a family meeting in a clear and compassionate manner

12. Listens to, encourages construction of, and documents care goals in collaboration with patients and family members

13. Prioritizes goals to achieve the outcomes deemed most important by patients and family members

14. Explains to and discusses with patients and family members why a particular treatment is inconsistent with the overall goals of care, using patients' preferences as a rubric for why the treatment is not appropriate.

15. Encourages and facilitates communication about conflicts between patients and family members regarding goals of care.

16. Uses family members as a source of information by verifying patient health history and medical, psychosocial, vocational, and financial condition

17. Assesses family members' health literacy and readiness to learn.

18. Assesses family members' current knowledge, received information and experience of family members regarding patients' diagnoses, treatments and prognosis.

19. Uses a range of strategies to communicate with family members, including reading, writing, speaking, validating, listening, teaching, and eliciting the stories of family members

20. Provides coherent and congruent information in easily understood language to keep the family members informed about diagnoses, treatments, progress, prognosis and transfers.

21. Communicates in an honest, compassionate, non-judgmental and calm manner to family members

22. Provides appropriate and timely information to patients and family members to facilitate understanding and support informed decision making

23. Discusses communication preferences with patients and family members

24. Provides care beyond technical-oriented tasks to connect with patients and family members in meaningful ways on a personal level.

25. Demonstrates respect for coping strategies and cultural and religious preferences and practices of patients and family members when discussing options, particularly when families decline evidence-based therapy

26. Establishes and maintains a therapeutic relationship with patients and family members.

4.1 Reflect about one's own process: Identify one's own expectations with regard to families in health and illness, including beliefs, values, attitudes, judgments, strengths, and limitations

4.2 Recognize that family nursing happens in relationships, which co-evolve through the nurse's and the family's contributions/interactions

4.3 Evaluate by asking for feedback from the family on success of intervention strategies and family progress to allow self-reflection on nurse activities

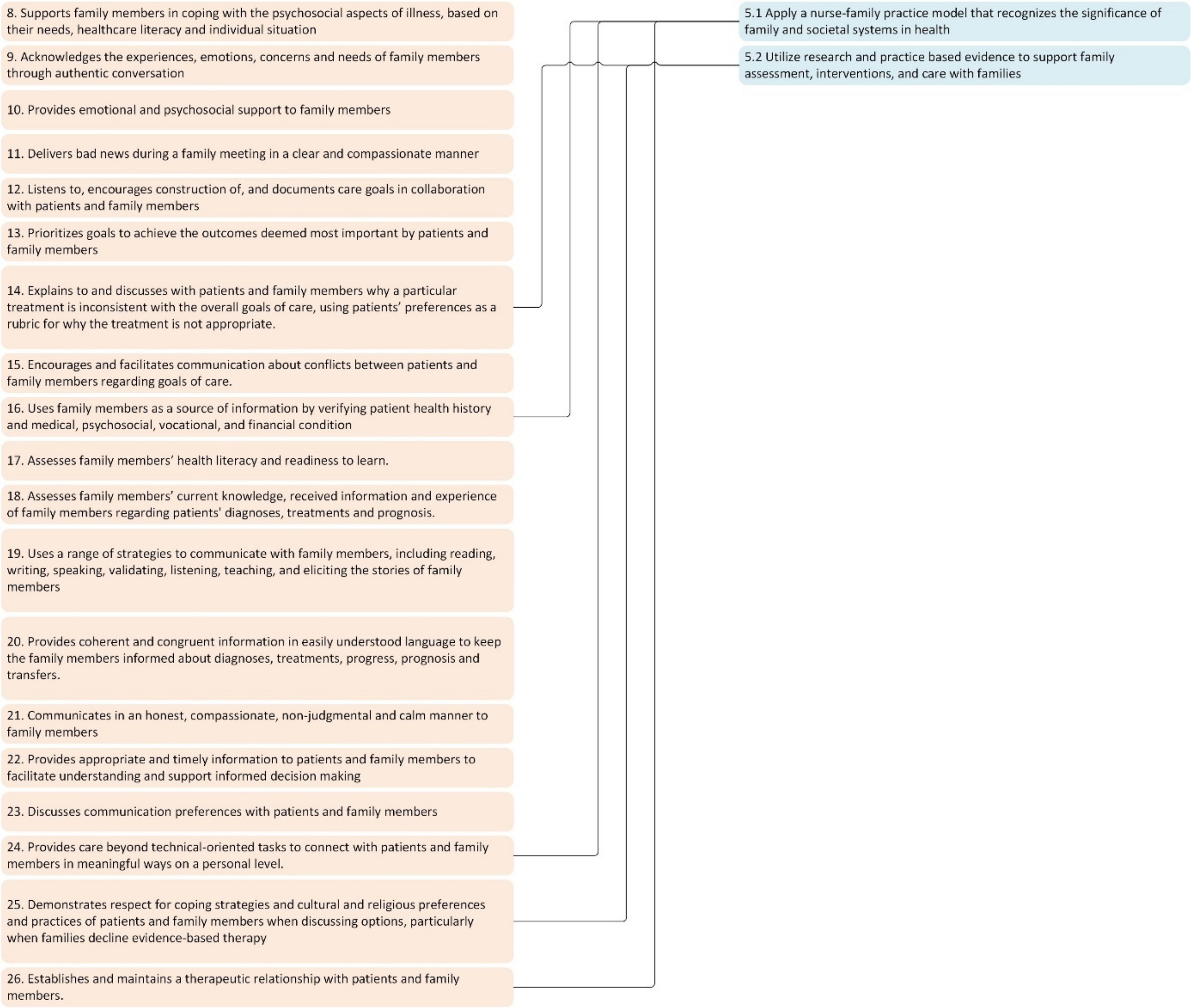
8. Supports family members in coping with the psychosocial aspects of illness, based on their needs, healthcare literacy and individual situation
9. Acknowledges the experiences, emotions, concerns and needs of family members through authentic conversation
10. Provides emotional and psychosocial support to family members
11. Delivers bad news during a family meeting in a clear and compassionate manner
12. Listens to, encourages construction of, and documents care goals in collaboration with patients and family members
13. Prioritizes goals to achieve the outcomes deemed most important by patients and family members
14. Explains to and discusses with patients and family members why a particular treatment is inconsistent with the overall goals of care, using patients' preferences as a rubric for why the treatment is not appropriate.
15. Encourages and facilitates communication about conflicts between patients and family members regarding goals of care.
16. Uses family members as a source of information by verifying patient health history and medical, psychosocial, vocational, and financial condition
17. Assesses family members' health literacy and readiness to learn.
18. Assesses family members' current knowledge, received information and experience of family members regarding patients' diagnoses, treatments and prognosis.
19. Uses a range of strategies to communicate with family members, including reading, writing, speaking, validating, listening, teaching, and eliciting the stories of family members
20. Provides coherent and congruent information in easily understood language to keep the family members informed about diagnoses, treatments, progress, prognosis and transfers.
21. Communicates in an honest, compassionate, non-judgmental and calm manner to family members
22. Provides appropriate and timely information to patients and family members to facilitate understanding and support informed decision making
23. Discusses communication preferences with patients and family members
24. Provides care beyond technical-oriented tasks to connect with patients and family members in meaningful ways on a personal level.
25. Demonstrates respect for coping strategies and cultural and religious preferences and practices of patients and family members when discussing options, particularly when families decline evidence-based therapy
26. Establishes and maintains a therapeutic relationship with patients and family members.
5.1 Apply a nurse-family practice model that recognizes the significance of family and societal systems in health
5.2 Utilize research and practice based evidence to support family assessment, interventions, and care with families

Collaborator:

Comparison of competencies in our set belonging to the CanMeds role of Collaborator (left) and the complete IFNA generalist set (right). We found no connection with domain 4 of the IFNA set.

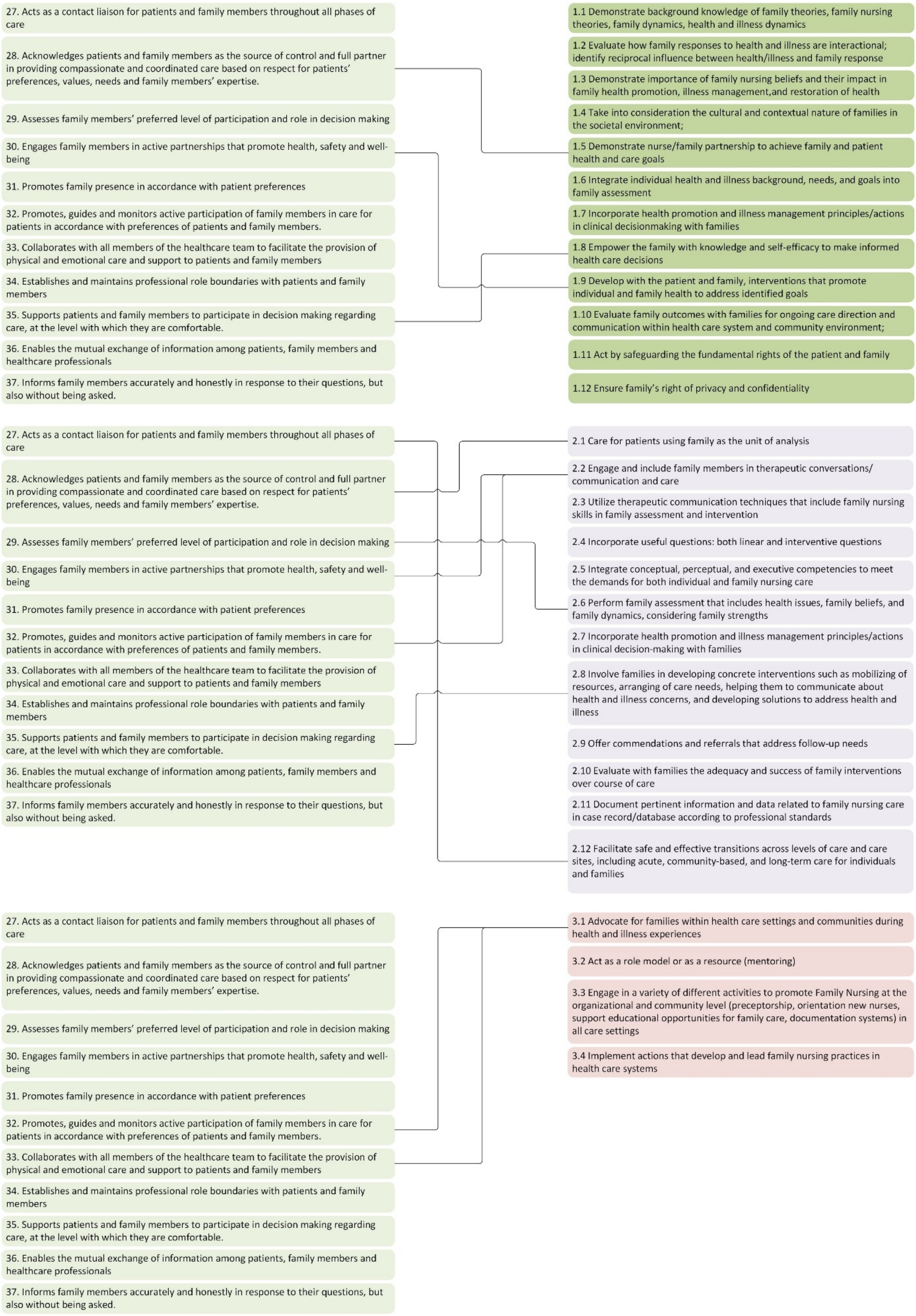

27. Acts as a contact liaison for patients and family members throughout all phases of care

28. Acknowledges patients and family members as the source of control and full partner in providing compassionate and coordinated care based on respect for patients' preferences, values, needs and family members' expertise.

29. Assesses family members' preferred level of participation and role in decision making

30. Engages family members in active partnerships that promote health, safety and well-being

31. Promotes family presence in accordance with patient preferences

32. Promotes, guides and monitors active participation of family members in care for patients in accordance with preferences of patients and family members.

33. Collaborates with all members of the healthcare team to facilitate the provision of physical and emotional care and support to patients and family members

34. Establishes and maintains professional role boundaries with patients and family members

35. Supports patients and family members to participate in decision making regarding care, at the level with which they are comfortable.

36. Enables the mutual exchange of information among patients, family members and healthcare professionals

37. Informs family members accurately and honestly in response to their questions, but also without being asked.

4.1 Reflect about one's own process: Identify one's own expectations with regard to families in health and illness, including beliefs, values, attitudes, judgments, strengths, and limitations

4.2 Recognize that family nursing happens in relationships, which co-evolve through the nurse's and the family's contributions/interactions

4.3 Evaluate by asking for feedback from the family on success of intervention strategies and family progress to allow self-reflection on nurse activities

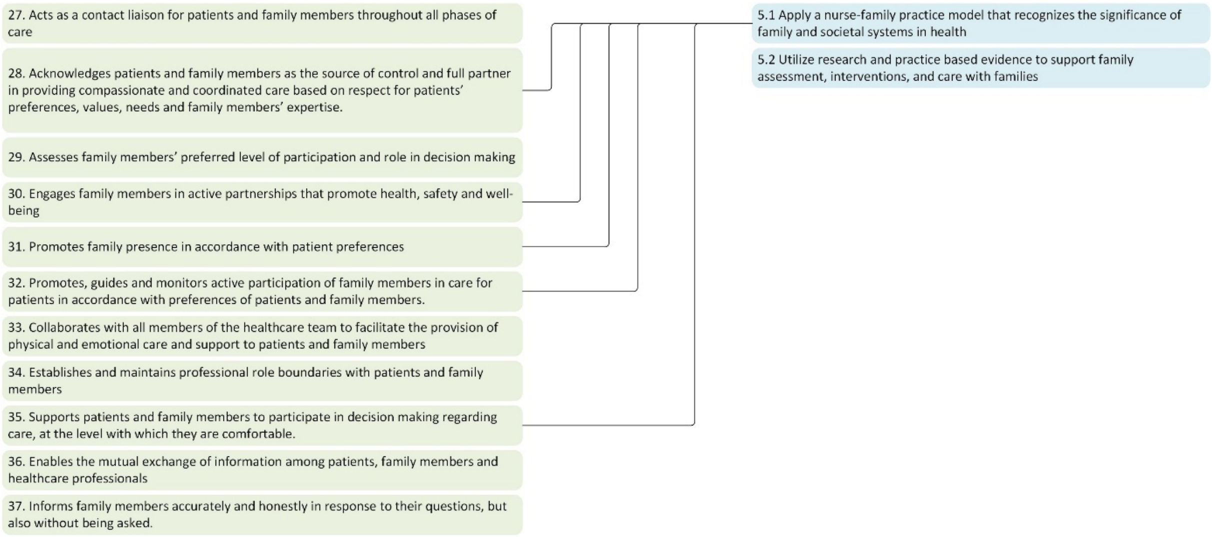

Leader:

Comparison of competencies in our set belonging to the CanMeds role of Leader (left) and the complete IFNA generalist set (right). We found no connection with domain 4 of the IFNA set.

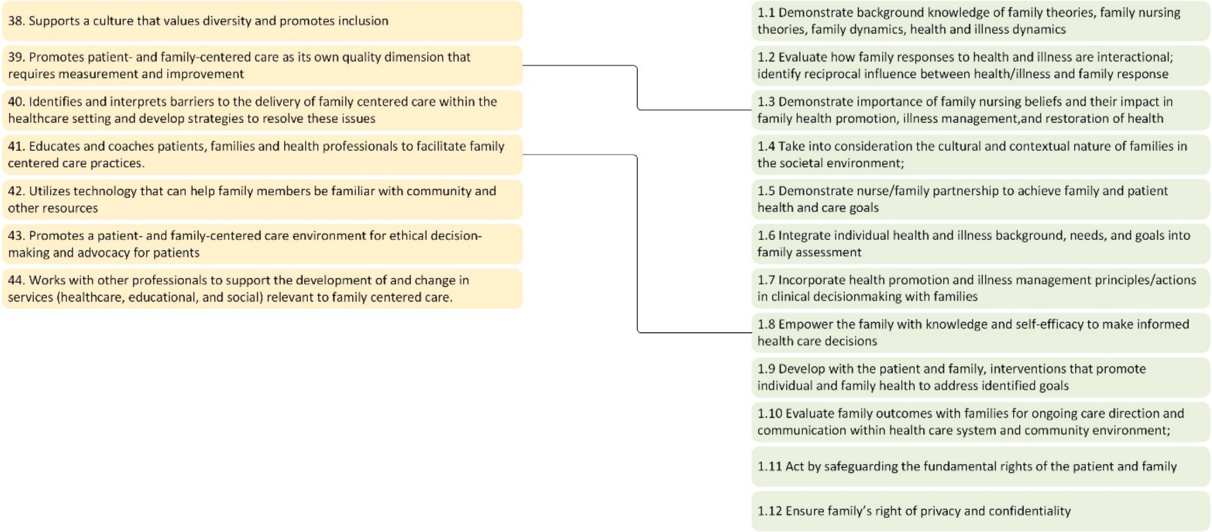

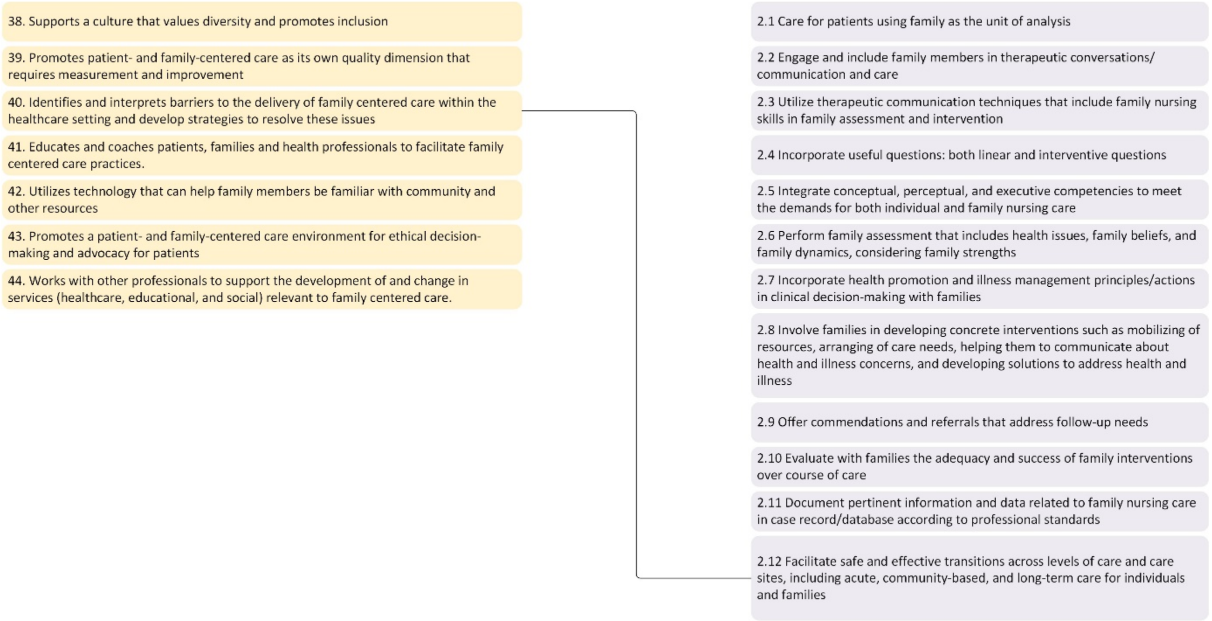

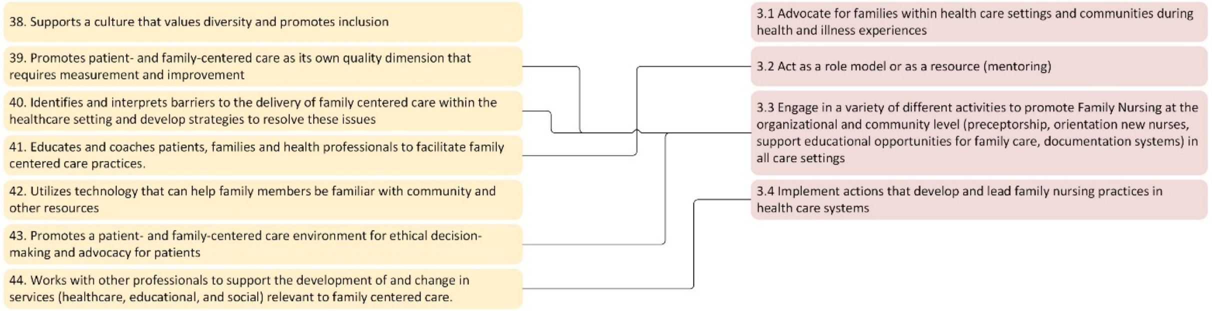

38. Supports a culture that values diversity and promotes inclusion

39. Promotes patient- and family-centered care as its own quality dimension that requires measurement and improvement

40. Identifies and interprets barriers to the delivery of family centered care within the healthcare setting and develop strategies to resolve these issues

41. Educates and coaches patients, families and health professionals to facilitate family centered care practices.

42. Utilizes technology that can help family members be familiar with community and other resources

43. Promotes a patient- and family-centered care environment for ethical decision-making and advocacy for patients

44. Works with other professionals to support the development of and change in services (healthcare, educational, and social) relevant to family centered care.

4.1 Reflect about one's own process: Identify one's own expectations with regard to families in health and illness, including beliefs, values, attitudes, judgments, strengths, and limitations

4.2 Recognize that family nursing happens in relationships, which co-evolve through the nurse's and the family's contributions/interactions

4.3 Evaluate by asking for feedback from the family on success of intervention strategies and family progress to allow self-reflection on nurse activities

38. Supports a culture that values diversity and promotes inclusion

39. Promotes patient- and family-centered care as its own quality dimension that requires measurement and improvement

40. Identifies and interprets barriers to the delivery of family centered care within the healthcare setting and develop strategies to resolve these issues

41. Educates and coaches patients, families and health professionals to facilitate family centered care practices.

42. Utilizes technology that can help family members be familiar with community and other resources

43. Promotes a patient- and family-centered care environment for ethical decision-making and advocacy for patients

44. Works with other professionals to support the development of and change in services (healthcare, educational, and social) relevant to family centered care.

5.1 Apply a nurse-family practice model that recognizes the significance of family and societal systems in health

5.2 Utilize research and practice based evidence to support family assessment, interventions, and care with families

Health advocate:

Comparison of competencies in our set belonging to the CanMeds role of Health advocatie (left) and the complete IFNA generalist set (right).

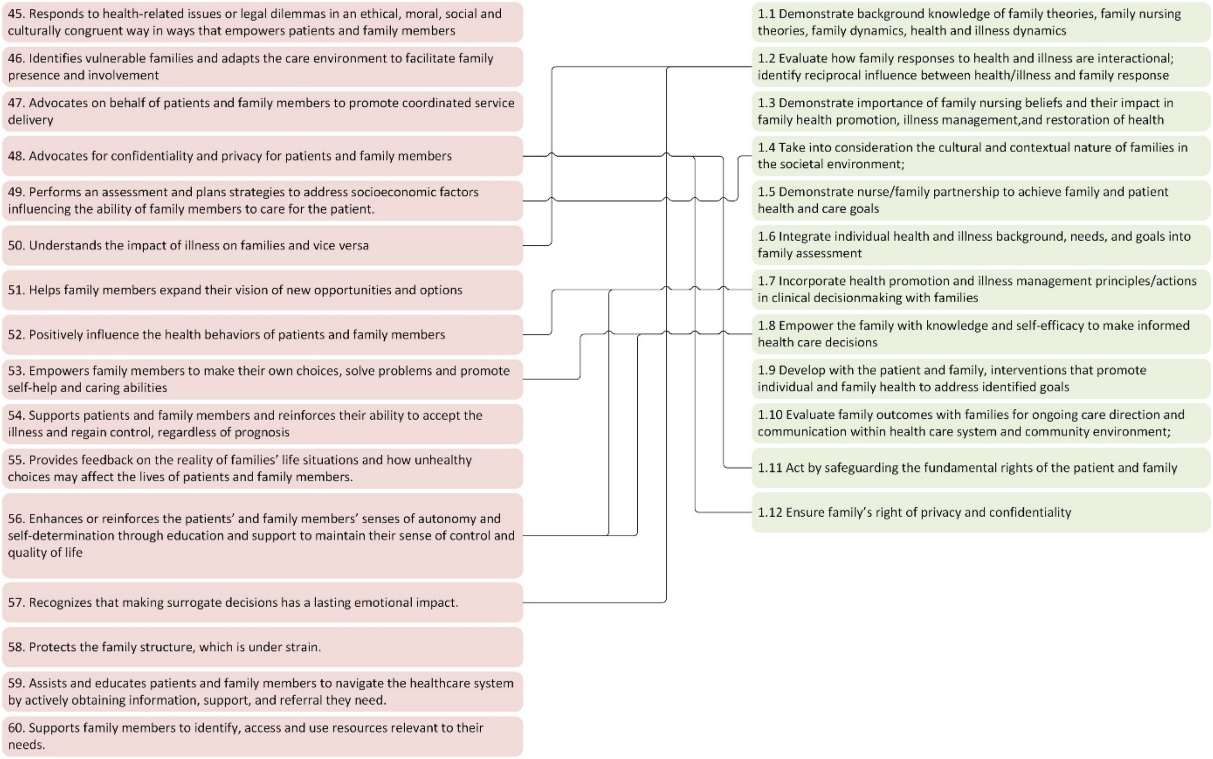

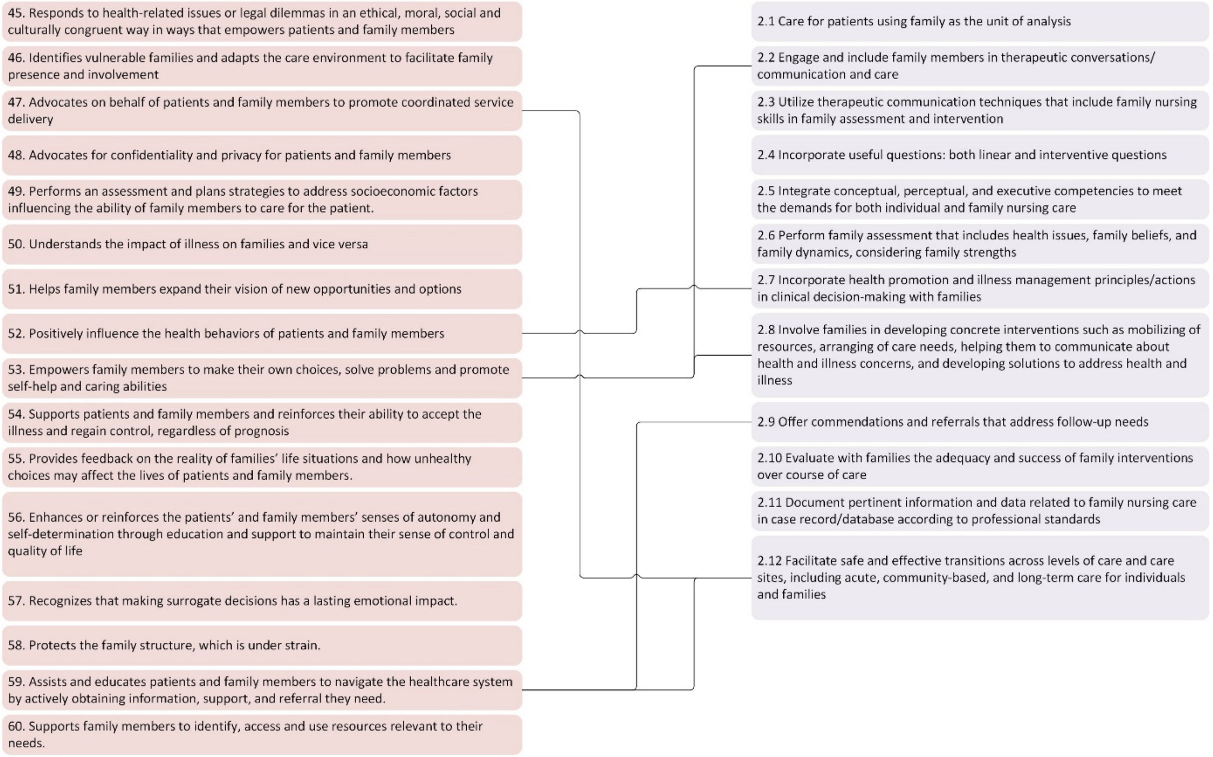

45. Responds to health-related issues or legal dilemmas in an ethical, moral, social and culturally congruent way in ways that empowers patients and family members

46. Identifies vulnerable families and adapts the care environment to facilitate family presence and involvement

47. Advocates on behalf of patients and family members to promote coordinated service delivery

48. Advocates for confidentiality and privacy for patients and family members

49. Performs an assessment and plans strategies to address socioeconomic factors influencing the ability of family members to care for the patient.

50. Understands the impact of illness on families and vice versa

51. Helps family members expand their vision of new opportunities and options

52. Positively influence the health behaviors of patients and family members

53. Empowers family members to make their own choices, solve problems and promote self-help and caring abilities

54. Supports patients and family members and reinforces their ability to accept the illness and regain control, regardless of prognosis

55. Provides feedback on the reality of families' life situations and how unhealthy choices may affect the lives of patients and family members.

56. Enhances or reinforces the patients' and family members' senses of autonomy and self-determination through education and support to maintain their sense of control and quality of life

57. Recognizes that making surrogate decisions has a lasting emotional impact.

58. Protects the family structure, which is under strain.

59. Assists and educates patients and family members to navigate the healthcare system by actively obtaining information, support, and referral they need.

60. Supports family members to identify, access and use resources relevant to their needs.

3.1 Advocate for families within health care settings and communities during health and illness experiences

3.2 Act as a role model or as a resource (mentoring)

3.3 Engage in a variety of different activities to promote Family Nursing at the organizational and community level (preceptorship, orientation new nurses, support educational opportunities for family care, documentation systems) in all care settings

3.4 Implement actions that develop and lead family nursing practices in health care systems

45. Responds to health-related issues or legal dilemmas in an ethical, moral, social and culturally congruent way in ways that empowers patients and family members

46. Identifies vulnerable families and adapts the care environment to facilitate family presence and involvement

47. Advocates on behalf of patients and family members to promote coordinated service delivery

48. Advocates for confidentiality and privacy for patients and family members

49. Performs an assessment and plans strategies to address socioeconomic factors influencing the ability of family members to care for the patient.

50. Understands the impact of illness on families and vice versa

51. Helps family members expand their vision of new opportunities and options

52. Positively influence the health behaviors of patients and family members

53. Empowers family members to make their own choices, solve problems and promote self-help and caring abilities

54. Supports patients and family members and reinforces their ability to accept the illness and regain control, regardless of prognosis

55. Provides feedback on the reality of families' life situations and how unhealthy choices may affect the lives of patients and family members.

56. Enhances or reinforces the patients' and family members' senses of autonomy and self-determination through education and support to maintain their sense of control and quality of life

57. Recognizes that making surrogate decisions has a lasting emotional impact.

58. Protects the family structure, which is under strain.

59. Assists and educates patients and family members to navigate the healthcare system by actively obtaining information, support, and referral they need.

60. Supports family members to identify, access and use resources relevant to their needs.

4.1 Reflect about one's own process: Identify one's own expectations with regard to families in health and illness, including beliefs, values, attitudes, judgments, strengths, and limitations

4.2 Recognize that family nursing happens in relationships, which co-evolve through the nurse's and the family's contributions/interactions

4.3 Evaluate by asking for feedback from the family on success of intervention strategies and family progress to allow self-reflection on nurse activities

45. Responds to health-related issues or legal dilemmas in an ethical, moral, social and culturally congruent way in ways that empowers patients and family members

46. Identifies vulnerable families and adapts the care environment to facilitate family presence and involvement

47. Advocates on behalf of patients and family members to promote coordinated service delivery

48. Advocates for confidentiality and privacy for patients and family members

49. Performs an assessment and plans strategies to address socioeconomic factors influencing the ability of family members to care for the patient.

50. Understands the impact of illness on families and vice versa

51. Helps family members expand their vision of new opportunities and options

52. Positively influence the health behaviors of patients and family members

53. Empowers family members to make their own choices, solve problems and promote self-help and caring abilities

54. Supports patients and family members and reinforces their ability to accept the illness and regain control, regardless of prognosis

55. Provides feedback on the reality of families' life situations and how unhealthy choices may affect the lives of patients and family members.

56. Enhances or reinforces the patients' and family members' senses of autonomy and self-determination through education and support to maintain their sense of control and quality of life

57. Recognizes that making surrogate decisions has a lasting emotional impact.

58. Protects the family structure, which is under strain.

59. Assists and educates patients and family members to navigate the healthcare system by actively obtaining information, support, and referral they need.

60. Supports family members to identify, access and use resources relevant to their needs.

5.1 Apply a nurse-family practice model that recognizes the significance of family and societal systems in health

5.2 Utilize research and practice based evidence to support family assessment, interventions, and care with families

Scholar

Comparison of competencies in our set belonging to the CanMeds role of Communicator (left) and the complete IFNA generalist set (right). We found no connection with domain 3 of the IFNA set.

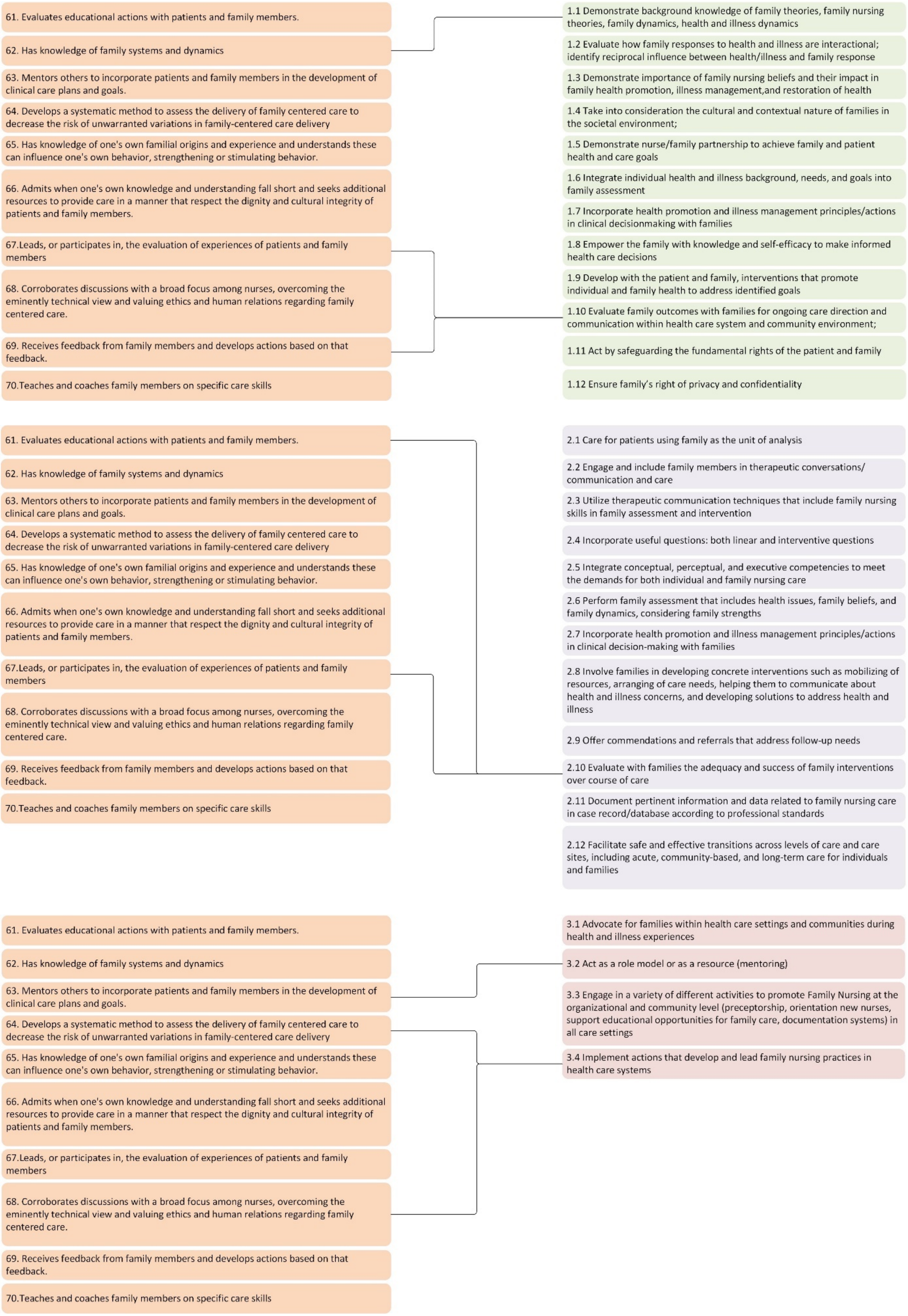

61. Evaluates educational actions with patients and family members.

62. Has knowledge of family systems and dynamics

63. Mentors others to incorporate patients and family members in the development of clinical care plans and goals.

64. Develops a systematic method to assess the delivery of family centered care to decrease the risk of unwarranted variations in family-centered care delivery

65. Has knowledge of one's own familial origins and experience and understands these can influence one's own behavior, strengthening or stimulating behavior.

66. Admits when one's own knowledge and understanding fall short and seeks additional resources to provide care in a manner that respect the dignity and cultural integrity of patients and family members.

67.Leads, or participates in, the evaluation of experiences of patients and family members

68. Corroborates discussions with a broad focus among nurses, overcoming the eminently technical view and valuing ethics and human relations regarding family centered care.

69. Receives feedback from family members and develops actions based on that feedback.

70.Teaches and coaches family members on specific care skills

4.1 Reflect about one's own process: Identify one's own expectations with regard to families in health and illness, including beliefs, values, attitudes, judgments, strengths, and limitations

4.2 Recognize that family nursing happens in relationships, which co-evolve through the nurse's and the family's contributions/interactions

4.3 Evaluate by asking for feedback from the family on success of intervention strategies and family progress to allow self-reflection on nurse activities

61. Evaluates educational actions with patients and family members.

62. Has knowledge of family systems and dynamics

63. Mentors others to incorporate patients and family members in the development of clinical care plans and goals.

64. Develops a systematic method to assess the delivery of family centered care to decrease the risk of unwarranted variations in family-centered care delivery

65. Has knowledge of one's own familial origins and experience and understands these can influence one's own behavior, strengthening or stimulating behavior.

66. Admits when one's own knowledge and understanding fall short and seeks additional resources to provide care in a manner that respect the dignity and cultural integrity of patients and family members.

67.Leads, or participates in, the evaluation of experiences of patients and family members

68. Corroborates discussions with a broad focus among nurses, overcoming the eminently technical view and valuing ethics and human relations regarding family centered care.

69. Receives feedback from family members and develops actions based on that feedback.

70.Teaches and coaches family members on specific care skills

5.1 Apply a nurse-family practice model that recognizes the significance of family and societal systems in health

5.2 Utilize research and practice based evidence to support family assessment, interventions, and care with families

Professional

Comparison of competencies in our set belonging to the CanMeds role of Communicator (left) and the complete IFNA generalist set (right). We found no connection with domains 4 and 5 of the IFNA set.

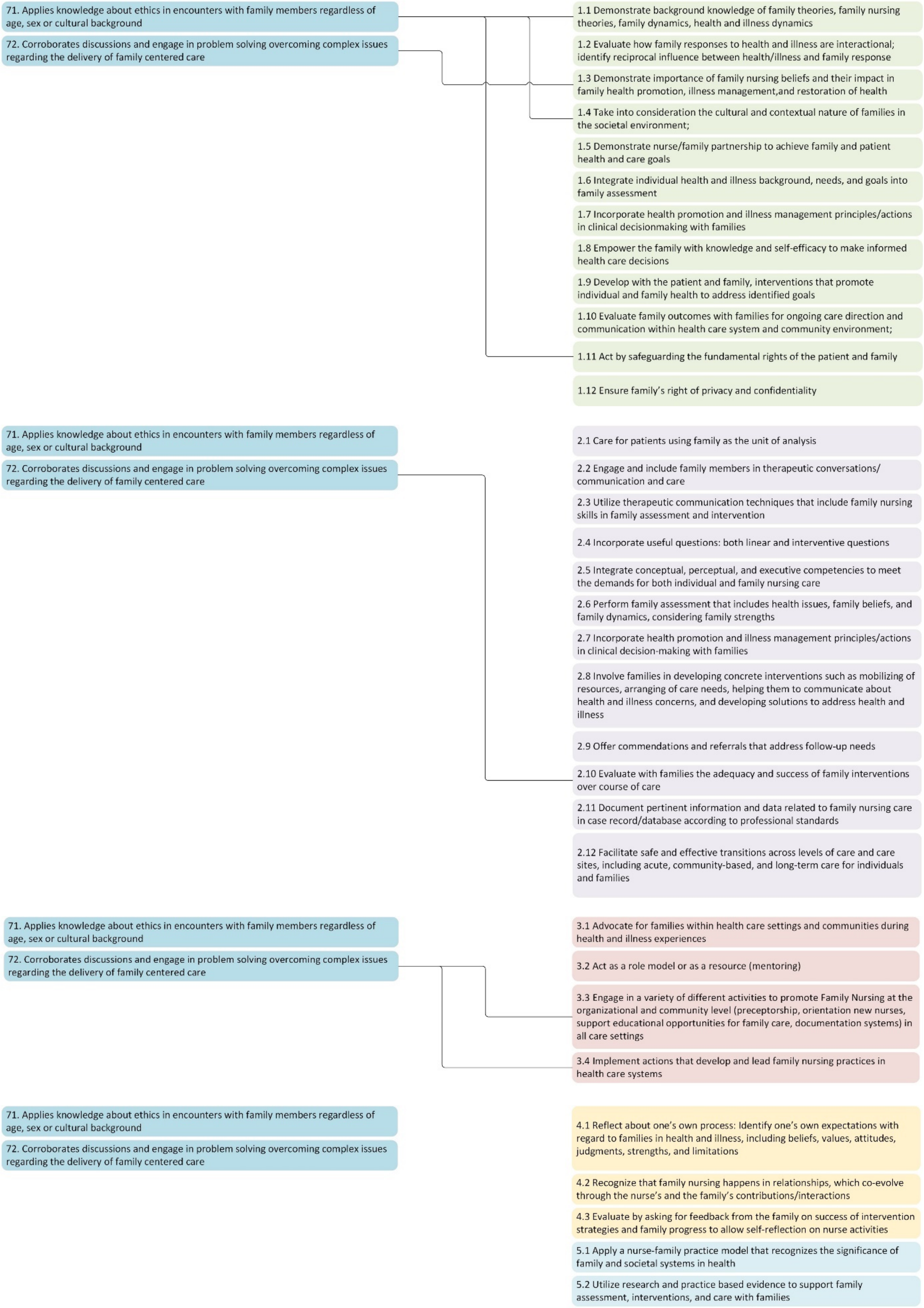

Supplementary file 7 - Unique competencies in our developed set

Competencies that are present in our newly developed set which we deemed not present in or markedly different in the IFNA set.

Competency	Role	Rank
4. Provides and reinforces education to patients and family members about diagnosis, treatment options, side effect management and post-treatment care	Nursing Expert	47
7. Assesses and evaluates the ability of families to deliver appropriate and safe care	Nursing Expert	37
8. Supports family members in coping with the psychosocial aspects of illness, based on their needs, healthcare literacy and individual situation	Communicator	26
10. Provides emotional and psychosocial support to family members	Communicator	20
11. Delivers bad news during a family meeting in a clear and compassionate manner	Communicator	55
13. Prioritizes goals to achieve the outcomes deemed most important by patients and family members	Communicator	9
17. Assesses family members’ health literacy and readiness to learn.	Communicator	34
18. Assesses family members’ current knowledge, received information and experience of family members regarding patients' diagnoses, treatments and prognosis.	Communicator	17
20. Provides coherent and congruent information in easily understood language to keep the family members informed about diagnoses, treatments, progress, prognosis and transfers.	Communicator	8
22. Provides appropriate and timely information to patients and family members to facilitate understanding and support informed decision making	Communicator	7
23. Discusses communication preferences with patients and family members	Collaborator	16
34. Establishes and maintains professional role boundaries with patients and family members	Collaborator	43
36. Enables the mutual exchange of information among patients, family members and healthcare professionals	Collaborator	18
37. Informs family members accurately and honestly in response to their questions, but also without being asked.	Collaborator	24
38. Supports a culture that values diversity and promotes inclusion	Leader	46
42. Utilizes technology that can help family members be familiar with community and other resources	Leader	71
45. Responds to health-related issues or legal dilemmas in an ethical, moral, social and culturally congruent way in ways that empowers patients and family members	Health Advocate	45
46. Identifies vulnerable families and adapts the care environment to facilitate family presence and involvement	Health Advocate	19
51. Helps family members expand their vision of new opportunities and options	Health Advocate	67
55. Provides feedback on the reality of families’ life situations and how unhealthy choices may affect the lives of patients and family members.	Health Advocate	70
57. Recognizes that making surrogate decisions has a lasting emotional impact.	Health Advocate	63
60. Supports family members to identify, access and use resources relevant to their needs.	Health Advocate	56
64. Develops a systematic method to assess the delivery of family centered care to decrease the risk of unwarranted variations in family-centered care delivery	Scholar	66
